# Single-site iron-anchored amyloid hydrogels as catalytic platforms for alcohol detoxification

**DOI:** 10.1038/s41565-024-01657-7

**Published:** 2024-05-13

**Authors:** Jiaqi Su, Pengjie Wang, Wei Zhou, Mohammad Peydayesh, Jiangtao Zhou, Tonghui Jin, Felix Donat, Cuiyuan Jin, Lu Xia, Kaiwen Wang, Fazheng Ren, Paul Van der Meeren, F. Pelayo García de Arquer, Raffaele Mezzenga

**Affiliations:** 1 Department of Health Sciences and Technology, ETH Zurich, Zurich, Switzerland; 2 Particle and Interfacial Technology Group, Faculty of Bioscience Engineering, Ghent University, Ghent, Belgium; 3 Department of Nutrition and Health, Beijing Higher Institution Engineering Research Center of Animal Products, China Agricultural University, Beijing, China; 4 Department of Chemistry and Applied Biosciences, ETH Zurich, Zurich, Switzerland; 5 Institute of Energy and Process Engineering, Department of Mechanical and Process Engineering, ETH Zurich, Zurich, Switzerland; 6 Institute of Translational Medicine, Zhejiang Shuren University, Zhejiang, China; 7 ICFO–Institut de Ciències Fotòniques, The Barcelona Institute of Science and Technology, Barcelona, Spain; 8 Department of Materials, ETH Zurich, Zurich, Switzerland

**Keywords:** Nanostructures, Nanoscale materials

## Abstract

Constructing effective antidotes to reduce global health impacts induced by alcohol prevalence is a challenging topic. Despite the positive effects observed with intravenous applications of natural enzyme complexes, their insufficient activities and complicated usage often result in the accumulation of toxic acetaldehyde, which raises important clinical concerns, highlighting the pressing need for stable oral strategies. Here we present an effective solution for alcohol detoxification by employing a biomimetic-nanozyme amyloid hydrogel as an orally administered catalytic platform. We exploit amyloid fibrils derived from β-lactoglobulin, a readily accessible milk protein that is rich in coordinable nitrogen atoms, as a nanocarrier to stabilize atomically dispersed iron (ferrous-dominated). By emulating the coordination structure of the horseradish peroxidase enzyme, the single-site iron nanozyme demonstrates the capability to selectively catalyse alcohol oxidation into acetic acid, as opposed to the more toxic acetaldehyde. Administering the gelatinous nanozyme to mice suffering from alcohol intoxication significantly reduced their blood-alcohol levels (decreased by 55.8% 300 min post-alcohol intake) without causing additional acetaldehyde build-up. Our hydrogel further demonstrates a protective effect on the liver, while simultaneously mitigating intestinal damage and dysbiosis associated with chronic alcohol consumption, introducing a promising strategy in effective alcohol detoxification.

## Abstract (web summary)

Oral antidotes for consumption-related acute alcohol intoxication are needed. Here, the study presents amyloid fibrils of β-lactoglobulin, a milk-derived protein decorated by single-site iron, as a catalytic platform for alcohol detoxification in vivo and prophylactic protection against alcohol damage.

## Main

Although widely enjoyed for its social and relaxing effects (Supplementary Fig. 1), alcohol consumption consistently poses significant risks to public health. In fact, in 2016 alone, harmful alcohol consumption resulted in nearly three million deaths and 132.6 million disability-adjusted life years^1–4^. Existing therapies, mainly relying on endogenous enzymes^5–7^, offer only temporary relief from symptoms, such as nausea and headaches, but fail to address other underlying issues, such as drowsiness, exhaustion and chronic alcoholism. Nanocomplexes with multiple complementary hepatic enzymes have emerged as an effective approach for accelerating human alcohol metabolism^8,9^. Although promising, a significant obstacle arises from the insufficient activity of commercially available enzymes, leading to the accumulation of a more hazardous intermediate, acetaldehyde, and possibly damage to human organs. Furthermore, natural enzymes possess major disadvantages, such as high cost, poor physicochemical stability and challenging storage, which have so far impeded the practical application of these complexes for alcohol detoxification purposes.

Over the past decades, advances in nanotechnology have facilitated the evolution of artificial enzymes into nanomaterials, that is, nanozymes, which have ignited enormous scientific interest across diverse fields, ranging from in vitro biosensing and detection to in vivo therapeutics^10–13^. Inspired by natural enzyme frameworks, researchers have predominantly focused on atomically distributed metal catalysts, in which the catalytic centre of natural enzymes is replicated at the atomic level^14–16^. These single-site catalysts, designed with well-defined electronic and geometric architectures, possess excellent catalytic capabilities, holding great potential as viable substitutes for natural enzymes. Given these promising prospects, attempts have been made to develop biomimetic nanozymes for alcohol detoxification by using, for example, natural enzymes on exogenous supports such as graphene oxide quantum dots or metal-organic framework nanozymes^17,18^. However, these approaches still either rely on natural enzymes or offer indirect effects, underscoring the potential for substantial design enhancements. The critical, yet challenging, aspect is the design of efficient single-site catalysts that are capable of converting ethanol into less-toxic acetic acid, or further into carbon dioxide and water, while minimizing the generation of acetaldehyde. Additionally, the task also lies in developing an orally administerable nanozyme that can withstand the gastrointestinal environment and which features no additional toxicity.

In this article, we report a biomimetic-nanozyme amyloid hydrogel to alleviate the deleterious effects of alcohol consumption via oral administration. Within this platform, single-site iron-anchored amyloid fibrils, an original kind of atomic-level engineered nanozyme featuring a similar coordination structure to horseradish peroxidase and with remarkable peroxidase-like activity, are used to efficiently catalyse alcohol oxidation. Specifically, the resultant nanozyme exhibits excellent selectivity in favour of acetic acid production. The catalytic activity of the gelatinous nanozyme could largely tolerate the digestive process, leading to a substantial decrease in blood alcohol levels in alcoholic mice, while avoiding the additional build-up of acetaldehyde. We finally demonstrate that this hydrogel also achieves heightened liver protection and substantial alleviation of intestinal damage and dysbiosis, thereby underscoring its potential as an improved therapeutic approach for alcohol-related conditions. By employing atomic-level design and harnessing the capabilities of nanozymes, our study offers promising insights into the development of efficient and targeted alcohol antidotes, with potential benefits for both liver protection and gastrointestinal health.

## Synthesis of single-site iron-anchored β-lactoglobulin fibrils

Diverging from conventional methods that use inorganic carriers, in the current work, we sought to utilize a readily available protein material, β-lactoglobulin (BLG) amyloid fibrils, as the supportive framework for atomically dispersed iron. In addition to their intrinsic binding affinity to various metal ions^19^, including iron, the large aspect ratio of protein filaments (Supplementary Fig. 2a) and tacked-up β-sheet units also enhance the accessibility of potential binding sites, thereby facilitating the high-density loading of iron atoms. Moreover, BLG fibrils can be easily derived from native BLG, a readily available milk protein, and have very recently been demonstrated safe nutrition ingredients by a comprehensive in vitro and in vivo assessment^20^, meeting the requirements for potential oral administration^21^. Moreover, the exceptional gelling property of BLG fibrils allows for the easy production of hydrogels^22^, which anticipates a delayed digestion process and a prolonged action time within the gastrointestinal tract due to their high viscoelasticity^23,24^.

**Fig. 1:**
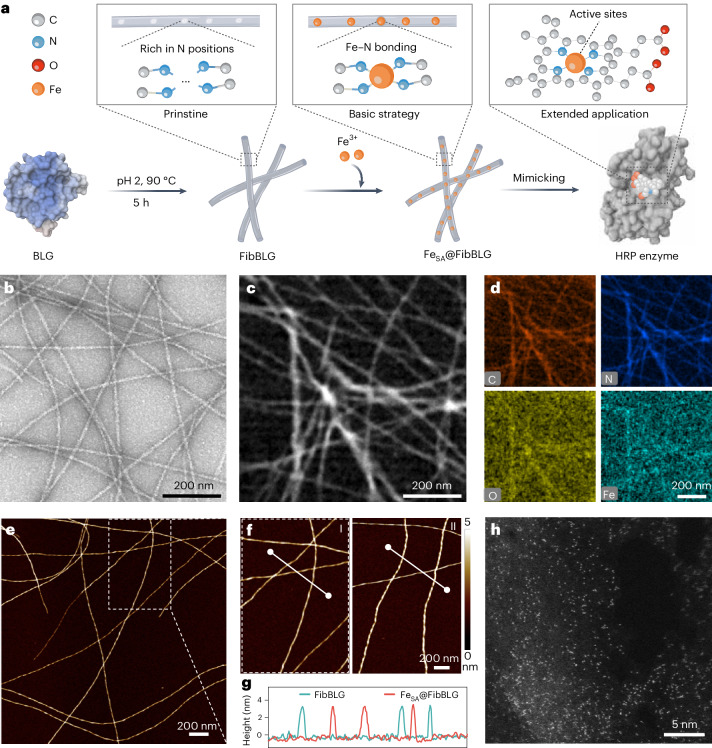
Synthesis and morphology characterization of Fe_SA_@FibBLG. **a**, Illustration of the synthesis process of Fe_SA_@FibBLG. **b**–**d**, TEM image (**b**), HAADF-STEM image (**c**) and the corresponding EDS mapping images (**d**) of Fe_SA_@FibBLG. **e**–**g**, AFM images of Fe_SA_@FibBLG (**e, f (I)**) and FibBLG (**f (II)**) on the mica surface and (**g**) the corresponding height profiles of the white auxiliary lines. **h**, Representative HAADF-STEM image of Fe_SA_@FibBLG. The images presented in **b**–**f**,**h** are representative of six technical replicates (*n* = 6), each yielding similar results. Source data

The single-site iron-anchored BLG fibrils (Fe_SA_@FibBLG) catalyst was synthesized by a straightforward wetness impregnation procedure (Fig. 1a), which involved exposing a dispersion of BLG fibrils in a mixture of ethanol and polyethylene glycol 200 (PEG200) to a Fe(NO_3_)_3_ PEG200 solution. During this process, the natural occurrence of nitrogen in BLG fibrils coordinated with iron ions to form functional Fe–N–C active sites. The resulting precipitate was lyophilized and collected after multiple rounds of centrifugation and washing.

Having synthesized Fe_SA_@FibBLG, we then performed a comprehensive characterization of the material using multiple analytical techniques. The morphology of Fe_SA_@FibBLG, which retains a nanometre-scale diameter consistent with pure BLG fibrils (Supplementary Fig. 2b), suggests minimal structural impact from the integration of iron (Fig. 1b and Supplementary Fig. 2b). The iron was homogeneously dispersed across the BLG fibril framework, as evidenced by a significant overlap of the Fe K-edge profile with the elemental composition of the BLG fibrils (Fig. 1c,d and Supplementary Fig. 2c). Atomic force microscopy (AFM) images confirmed a consistent height of approximately 3 nm both before and after iron integration, verifying the negligible presence of crystalline iron or oxide species (Fig. 1e,f,g). As shown in Fig. 1h and Supplementary Fig. 2d–f, the presence of individual bright dots with a size below 0.2 nm clearly demonstrated the atomic dispersion of single iron atoms over Fe_SA_FibBLG, indicating that iron, upon participating in the synthetic procedure described above, is present exclusively in single-site form on the BLG fibrils.

## Structural analysis of Fe_SA_@FibBLG

**Fig. 2:**
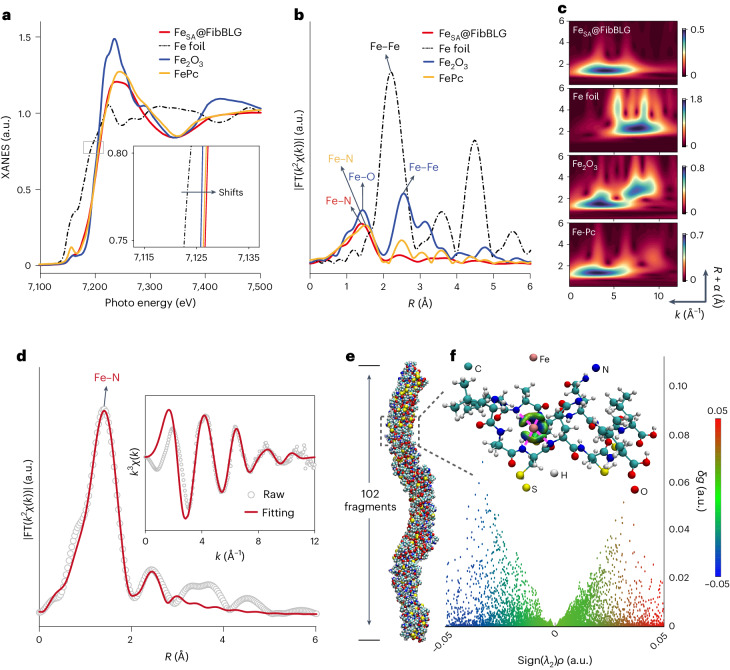
Atomic structure characterization of Fe_SA_@FibBLG. **a**, Normalized XANES spectra at the Fe K-edge of Fe_SA_@FibBLG along with reference samples. **b**, Fourier-transformed (FT) magnitudes of the experimental Fe K-edge EXAFS signals of Fe_SA_@FibBLG along with reference samples. **c**, Wavelet transform analysis of Fe K-edge EXAFS data. **d**, Fitting curves of the EXAFS of _FeSA@FibBLG_in the *R*-space and *k*-space (inset). Fitting results are summarized in Supplementary Table 1. **e**, Representative snapshots of the assembly structure of 102 amyloid-forming fragments (LACQCL) from BLG in the process of AAMD simulation using the Gromacs54A force field at 10 ns. **f**, The 3D gradient isosurfaces and corresponding 2D scatter diagram of *δg* versus sign(*λ*_2_)ρ for possible non-covalent interactions between a single iron atom and dimer intercepted from BLG fibril segments in **e** through DFT simulation. *δg* is a quantitative measure derived from comparing electron density gradients in the presence and absence of interference, highlighting the penetration of electron density from one Bader atom to its neighbor; sign(λ_2_)*ρ* is a scalar field value used to describe the product of the sign of the second eigenvalue (λ_2_) of the Hessian matrix of a scalar field and the scalar field’s density (*ρ*). Source data

The coordination environment of iron within Fe_SA_@FibBLG was elucidated by X-ray absorption fine structure (XAFS) spectroscopy^25^. Figure 2a shows that the pre-edge position for Fe_SA_@FibBLG resided between the positions of iron foil (metallic iron) and Fe_2_O_3_. The white line area located at higher binding energy demonstrates a lower oxidation state and different coordination environments compared with Fe_2_O_3_ (ref. ^26^). X-ray absorption near-edge spectroscopy (XANES) features are valuable for discerning site symmetry around iron in macromolecular complexes^27^. A distinct prominent pre-edge feature below 7,120 eV indicates the ferrous iron (Fe^2+^) square-planar coordination in iron(II) phthalocyanine (FePc), whereas in Fe_SA_@FibBLG this feature is slightly reduced due to deviations from ideal square-planarity^28^. The XANES spectrum of Fe_SA_@FibBLG (Fig. 2a, inset) closely resembles that of FePc, implying a positively charged ionic state of iron within Fe_SA_@FibBLG (Fe^*δ*+^, where the average *δ* is close to 2). Further insights were obtained from extended X-ray absorption fine structure (EXAFS) spectra in *R*-space (Fig. 2b), which revealed a single peak at approximately 1.4 Å. From comparison with reference materials this peak was attributable to the backscattering between iron and lighter atoms, primarily nitrogen (Fig. 2b), supporting the atomic dispersion of iron sites within Fe_SA_@FibBLG. Wavelet transform analysis differentiated the sample from the iron foil reference by showing a single maximum intensity at approximately 4 Å^−1^ and 1.4 Å, suggesting significant Fe–N contributions (Fig. 2c and Supplementary Fig. 3), with the coordination number of iron estimated to be 4.5 (Fig. 2d and Supplementary Table 1). However, given the challenge in distinguishing Fe–N from Fe–O coordination compared to references such as FePc and Fe_2_O_3_, it is crucial to emphasize the potential existence of Fe–O bonds. Collectively, these findings confirmed that iron in Fe_SA_@FibBLG exists as single-site iron, devoid of any crystalline or oxide iron metal structure and mainly coordinates with nitrogen atoms. X-ray photoelectron spectroscopy (XPS) analysis of Fe_SA_@FibBLG further identified distinct binding states of carbon, nitrogen, oxygen and iron, demonstrating a majority of single-site iron in the Fe^2+^ state and the existence of Fe–N coordination (Supplementary Figs. 4 and 5)^29–31^.

Next, we performed a density functional theory (DFT) calculation for the process of anchoring a ferric ion onto the BLG fibril structure. Since the formation of BLG fibrils involved the participation of multiple peptides assembling in a random manner, here a model nanofibre structure was generated in silico based on repetitive amyloid-forming fragments (LACQCL) from BLG, using an all-atom molecular dynamics (AAMD) simulation (Fig. 2e)^19^. An evident periodic nanofibril was formed at 10 ns containing 102 repetitive fragments, where a peptide dimer with verified thermodynamic stability was intercepted for DFT calculation (Supplementary Fig. 6). As shown in Fig. 2f, the blue isosurface observed between the iron atom and surrounding nitrogen atoms corresponds to strong attractive interactions between iron and nitrogen, potentially arising from the sharing of electron pairs between the iron and nitrogen atoms (Supplementary Fig. 7). This was further verified by the existence of the prominent peak at approximately −0.03 in the scatter plot (Fig. 2f). These results clearly demonstrate that the BLG fibrils possessed effective binding sites that were capable of capturing iron atoms through Fe–N coordination, enabling the formation of active iron centres in Fe_SA_@FibBLG.

## Peroxidase-like activity of Fe_SA_@FibBLG

**Fig. 3:**
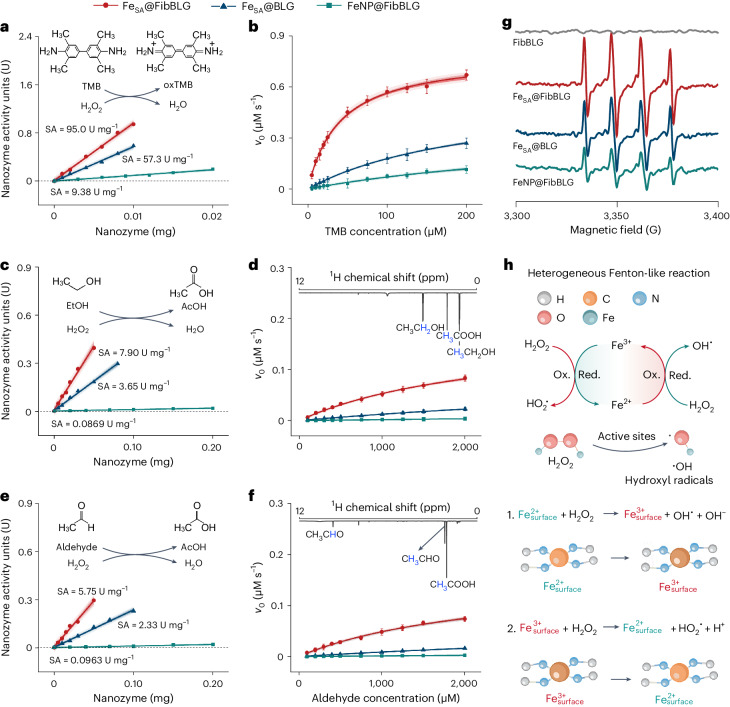
Peroxidase-like activity of single-site iron-anchored BLG fibrils. **a**–**f**, Typical Michaelis–Menten curves of Fe_SA_@FibBLG, Fe_SA_@BLG and FeNP@FibBLG by varying the TMB (**a**), ethanol (**c**) and acetaldehyde (**e**) concentrations in the presence of H_2_O_2_. Comparison of the SAs (U mg^−1^) of Fe_SA_@FibBLG, Fe_SA_@BLG and FeNP@FibBLG on TMB (**b**), ethanol (**d**) and acetaldehyde (**f**) oxidation in the presence of H_2_O_2_. One nanozyme activity unit (U) is defined as the amount of nanozyme that catalyses 1 µmol of product per minute. The SAs (U mg^−1^) were determined by plotting the nanozyme activities against their weight and measuring the gradients of the fitting curves. ^1^H NMR spectrum of the reaction products of Fe_SA_@FibBLG-catalysed ethanol (inset **d**) and acetaldehyde (inset **f**) oxidation. Data are presented as the mean ± s.d. from *n* = 3 independent experiments. **g**, EPR spectra of 5,5-dimethyl-pyrroline-*N*-oxide/H_2_O_2_ solution upon the addition of nanozymes. **h**, Schematic illustration of the peroxidase-like activities of Fe_SA_@FibBLG when exposed to various substrates. Source data

The coordination structure of the catalytic sites in our Fe_SA_@FibBLG was similar to that of the horseradish peroxidase enzyme (Supplementary Fig. 8a)^32^. Inspired by this similarity, we characterized the peroxidase-like activities of Fe_SA_@FibBLG by studying the facilitated chromogenic reactions through catalysing artificial substrates of peroxidase (for example, 3,3′,5,5′-tetramethylbenzidine (TMB), 2,2′-azino-bis(3-ethylbenzothiazoline-6-sulfonic acid) or *o*-phenylenediamine) in the presence of H_2_O_2_ (Supplementary Fig. 8b). By using the general method described in the current work, two comparison catalysts, namely, single-site iron-anchored BLG (Fe_SA_@BLG), and iron-nanoparticle-anchored BLG fibrils (FeNP@FibBLG), were synthesized and then used to characterize the enzymatic activity (Supplementary Figs. 9 and 10 and Supplementary Table 2). Using TMB as a substrate, the specific activity (SA) values (U mg^−1^) of these nanozymes were measured: the SA of Fe_SA_@FibBLG was markedly superior, at 95.0 U mg^−1^, approximately 1.7 and 10.1 times higher than the SAs of Fe_SA_@BLG (57.3 U mg^−1^) and FeNP@FibBLG (9.38 U mg^−1^), respectively (Fig. 3a). Steady-state kinetic assays revealed that Fe_SA_@FibBLG exhibited superior catalytic performance among the tested nanozymes in oxidizing TMB, with remarkable kinetic parameters including maximum reaction rate (*V*_max_ = 0.788 μM s^−1^), turnover number (*K*_cat_ = 21.9 min^−1^), catalytic efficiency (*K*_cat_/*K*_m_ = 5.47 × 10^8^ M^−1^ min^−1^) and selectivity (*K*_m_ = 4.00 × 10^–2^ mM) (Fig. 3b and Supplementary Table 3). We also determined the kinetic parameters for the H_2_O_2_ substrate, which further substantiated the exceptional catalytic performance of Fe_SA_@FibBLG (Supplementary Table 4).

Interestingly, Fe_SA_@FibBLG also exhibited a notable capacity for catalytically oxidizing ethanol and acetaldehyde in the presence of H_2_O_2_ (Fig. 3c–f). The SA of Fe_SA_@FibBLG achieved a value of 7.90 U mg^−1^ when ethanol was used as the substrate, remarkably surpassing the other two reference catalysts. The superior catalytic efficacy of Fe_SA_@FibBLG with respect to ethanol was further confirmed by determining its kinetic parameters, which indicate it achieves a catalytic efficiency (*K*_cat_/*K*_m_ = 4.11 × 10^5^ M^−1^ min^−1^) that exceeds that of Fe_SA_@BLG (*K*_cat_/*K*_m_ = 8.66 × 10^4^ M^−1^ min^−1^) by 4.7 times and FeNP@FibBLG (*K*_cat_/*K*_m_ = 9.25 × 10^3^ M^−1^ min^−1^) by 44.4 times (Supplementary Table 5). Fe_SA_@FibBLG also manifested the lowest *K*_m_ value when ethanol was the substrate, signifying its excellent affinity towards ethanol. It is important to note that Fe_SA_@FibBLG could directly oxidize ethanol to acetic acid, yielding formic acid as the only by-product, without generating any detectable acetaldehyde intermediate, as evidenced by ^1^H NMR (Fig. 3d, inset).

To explain this, we performed a steady-state kinetic analysis of Fe_SA_@FibBLG participating in acetaldehyde oxidation. We found Fe_SA_@FibBLG to have the lowest *K*_m_ value of the evaluated nanozymes, signifying its superior substrate affinity towards acetaldehyde. The *K*_cat_/*K*_m_ for this reaction (3.89 × 10^5^ M^−1^ min^−1^) was very close to that for ethanol oxidation (4.11 × 10^5^ M^−1^ min^−1^) (Supplementary Tables 5 and 6). Upon the reaction between these nanozymes and H_2_O_2_, the electron paramagnetic resonance (EPR) spectrum exhibited characteristic peaks associated with 5,5-dimethyl-pyrroline-*N*-oxide–OH^**·**^, with Fe_SA_@FibBLG displaying the strongest EPR signal, indicating the highest production of OH^**·**^ (Fig. 3g). The same characteristic peaks were observed in the EPR spectrum of the Fe_SA_@FibBLG/H_2_O_2_/ethanol reaction system (Supplementary Fig. 17), confirming the existence of OH^**·**^ in ethanol oxidation—a finding that agrees with numerous studies demonstrating the efficacy of OH^**·**^ in oxidizing diverse organic compounds, including ethanol and acetaldehyde^33,34^. Nevertheless, it is essential to emphasize that our investigation serves as a preliminary exploration of the free radicals involved in this reaction; a more comprehensive mechanistic investigation is required for an in-depth understanding of the catalytic process.

Additionally, the catalytic stability of Fe_SA_@FibBLG was assessed by high-resolution transmission electron microscopy (TEM), high-angle annular dark-field scanning transmission electron microscopy (HAADF-STEM), energy-dispersive spectroscopy elemental analysis, X-ray diffraction and XPS (Supplementary Figs. 18 and 19). Fe_SA_@FibBLG did not exhibit substantial morphological or oxidation state alterations and effectively preserved the high atomic dispersion of iron active sites throughout the catalysis. It is also worth mentioning that Fe_SA_@FibBLG retained at least 95.2% and 84.1% of its activity after undergoing 3 h of digestion in simulated gastric and intestinal fluids, respectively (Supplementary Fig. 20). The robust stability observed in Fe_SA_@FibBLG may be due to the reduction effects of BLG fibril support^21^.

## Protective potential on acute alcohol intoxication

**Fig. 4:**
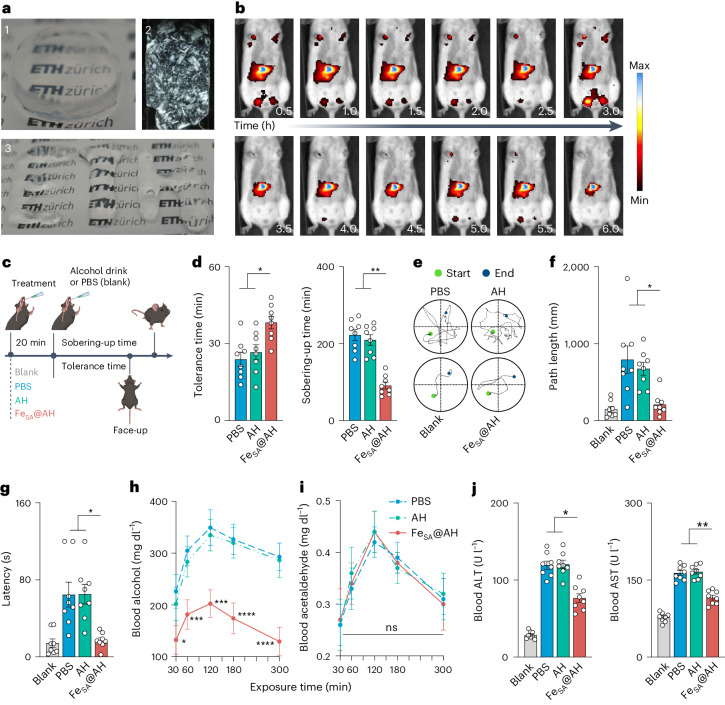
Effect of Fe_SA_@AH as a prophylactic for acute alcohol intoxication. **a**, Visualization (1) and microstructures (2) of Fe_SA_@AH under polarized light, and injectability test (3). **b**, Time-series images of gastrointestinal translocation of [^18^F]FDG-loaded Fe_SA_@AH in mice (0–6 h). **c**, Schematic of acute alcohol intoxication model construction (Methods). Created with BioRender.com. **d**, Effect of different treatment (PBS, AH and Fe_SA_@AH) on alcohol tolerance time and sobering-up time in C57BL/6 mice. **e**, Representative trajectory of search strategies of mice with different treatments. **f**,**g**, Escape latencies (**f**) and path length (**g**) of four groups of mice. **h**,**i**, Mean concentrations of blood alcohol (**h**) and acetaldehyde (**i**) in alcohol-intoxicated mice treated with PBS, AH and Fe_SA_@AH. **j**, Serum levels of ALT and AST enzyme levels in four groups of mice. Data are obtained for *n* = 8 independent biological replicates, mean ± s.e.m. *P* values in **d**,**f**,**g**,**h**,**j** were tested by one-way analysis of variance followed by Tukey–Kramer test. *P < 0.05, **P < 0.01, ***P < 0.001, ****P < 0.0001. Source data

Even a single new onset of blood alcohol that exceeds the detoxifying capability of the hepatic system can induce individual symptoms of acute alcohol intoxication, such as hepatocyte destruction, stress response and cognitive deficits^35,36^. To mitigate potential damage to the human digestive tract from direct H_2_O_2_ ingestion, a biomimetic cascade catalysis system was designed by integrating gold nanoparticles (AuNPs) for onsite and sustainable H_2_O_2_ generation^37–39^. AuNPs have demonstrated exceptionally efficient and enduring catalytic activity similar to glucose oxidase, which allows the conversion of glucose into gluconic acid, accompanied by the production of adequate H_2_O_2_ (Supplementary Fig. 21). Because protein fibrils transiently remained and were mostly digested (generally within 4 h) in the gastrointestinal tract^20^, where the majority of alcohol was absorbed, a salt-induced technique^40^ (Methods) was followed to fabricate the AuNP-attached Fe_SA_@FibBLG amyloid hydrogel (Fe_SA_@AH) (Supplementary Fig. 22) to achieve prolonged retention within the gastrointestinal tract, and, thereby, an enhanced overall capacity for ethanol oxidation. The resultant Fe_SA_@AH showed typical self-standing ability, obvious nanofibril structures (exceptional birefringence under polarized light) and good syringability (Fig. 4a). We then labelled Fe_SA_@AH with [^18^F]fluoro-2-deoxyglucose ([^18^F]FDG) and visualized its transportation in C57BL/6 mice by using micro positron emission tomography (PET)–computed tomography (CT) scanning. The metabolism of Fe_SA_@AH took more than 6 h in the upper gastrointestinal tract after gavage, which indicated an extended retention time in vivo due to the hydrogel nature of the compound^20^.

The prophylactic benefits of Fe_SA_@AH administration were assessed in an alcohol-treated murine model^41^ (Fig. 4c). A group of ethanol-free, but PBS-gavaged mice served as a negative control; all the ethanol-gavaged mice were asleep for alcohol intoxication. Although they tolerated alcohol intake for a longer period of time (∼40 min), the Fe_SA_@AH mice were awoken significantly earlier (∼2 h) than other intoxicated groups (Fig. 4d). We then conducted the Morris water maze (MWM) test 6 h post-alcohol intake to quantitatively assess murine spatial reference memory (Fig. 4e). Grouped mean swimming speeds of alcohol-exposed mice were comparable to those of the blank group, indicating recovery of fundamental activities (Supplementary Fig. 25a). However, PBS- and AH-treated mice showed increased search time and distance to locate the hidden platform, whereas the mice given Fe_SA_@AH demonstrated markedly improved navigational efficiency (Fig. 4f,g). Additionally, distinct search strategies were observed, with PBS and AH groups favouring less efficient patterns, in contrast to the strategic approaches of the Fe_SA_@AH and control groups (Supplementary Fig. 25b).

Aetiologically, behavioural abnormalities were attributed to alcohol and its in vivo intermediate metabolite, acetaldehyde^42^, and the liver played a core role in ethanolic metabolism. Prophylactic Fe_SA_@AH immediately and persistently reduced the mice blood alcohol (BA) concentration by a significant amount (Fig. 4h). The BA in Fe_SA_@AH mice decreased by 41.3%, 40.4%, 42.0%, 46.6% and 55.8%, respectively, 30, 60, 120, 180 and 300 min post-gavaging. Importantly, the above-mentioned process induced no additional acetaldehyde (BAce) accumulation in blood (Fig. 4i), which plays a crucial role in safeguarding the liver, as the build-up of acetaldehyde is known to be a catalyst for liver cirrhosis and hepatocellular carcinoma. Stress responses of liver were definitely mitigated, which was revealed by the significantly decreased blood alanine aminotransferase (ALT), aspartate aminotransferase (AST), malondialdehyde (MDA) and glutathione (GSH) levels in the Fe_SA_@AH group (Fig. 4j and Supplementary Fig. 26a,b).

## Prophylactic effect on chronic alcohol intoxication

**Fig. 5:**
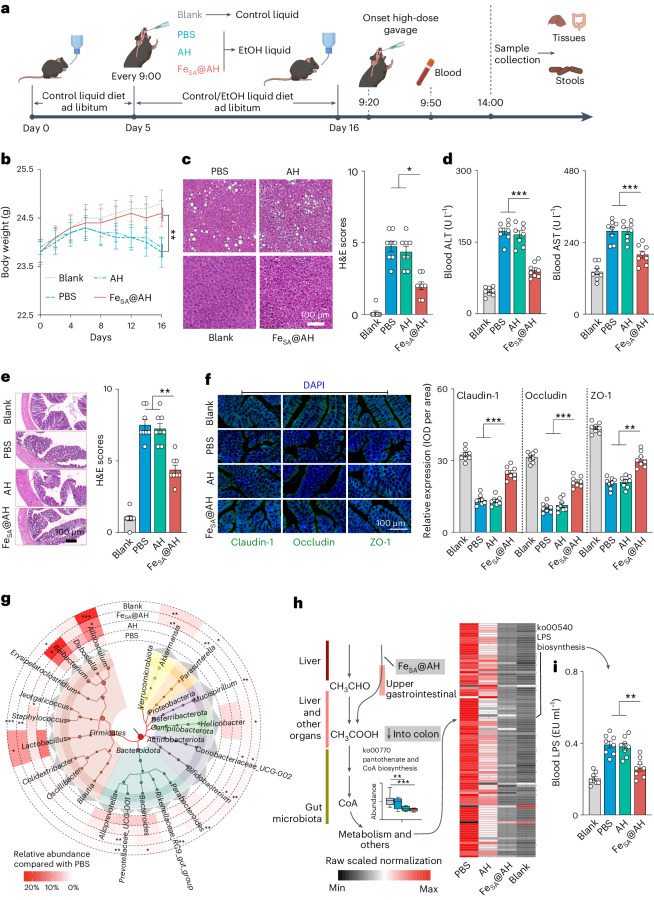
Effect of Fe_SA_@AH as a prophylactic for chronic alcohol intoxication. **a**, Schematic of the chronic alcohol intoxication model construction (Methods). Created with BioRender.com. **b**, Body weight changes in the four groups of mice during the feeding period. **c**, Representative H&E-stained images of liver in the four groups. **d**, Serum ALT and AST levels in mice. **e**, H&E images of colon (left part) and its assessed scores (right histogram) in different groups of mice. **f**, Immunofluorescence staining of the tight junction proteins in the colon (left part, 30× magnification). The tight junction proteins (Claudin-1, occludin and ZO-1) were stained green whereas the 4,6-diamidino-2-phenylindole (DAPI) was blue. The histograms (right) show the mean density of the normalized levels of occludin and ZO-1. IOD, integrated optical density. **g**, Taxonomic and phylogenetic tree of the top 21 most affected genera (genus with >10% mean abundance change in at least one group compared to others) by different treatments generated by GraPhlAn 4.0. Outer circles show the grouped mean relative abundance of each genus. **h**, Metabolic processes of alcohol to acetate and further in mice. The left colour blocks indicate the endogenous organs, liver, intestine and gut microbiota involved in alcohol decomposition, and the right shows the path in which Fe_SA_@AH participated. The box-plot shows the relative levels of ko00770 pantothenate and CoA biosynthesis among groups (minimum–maximum). The heatmap shows 83 significantly changed pathways compared with those in the PBS group. Source data are provided as a Source Data file. **i**, LPS concentrations of mice in the four groups. Data are shown in the form of mean ± s.e.m. from *n* = 8 biological replicates. In **c**,**e**,**f**, the images displayed are representative of three independent biological replicates (*n* = 3), each producing consistent results. For histopathological, physiological and biochemical indexes (**c**–**f**,**i**), *P* values were tested by one-way analysis of variance followed by Tukey–Kramer test whereas the pairwise Wilcoxon test with Bonferroni–Holm correction was used for microbial taxa (**h**,**i**). *P < 0.05, **P < 0.01, ***P < 0.001. Source data

The NIAAA model (mouse model of chronic and binge ethanol feeding) was conducted to confirm the long-term beneficial effects of Fe_SA_@AH^43^. After model constructions (Fig. 5a and Methods), the PBS mice showed a significantly decreased body weight, increased liver injury (ballooning degeneration and multifocal inflammatory cell infiltration) and hepatic lipid accumulation compared with the blank (Fig. 5b,c). Notably, Fe_SA_@AH-rescued mice showed a significantly decreased loss in body weight, less liver damage and re-regulated hepatic lipid metabolism (Fig. 5b,c) from intoxication. Moreover, mice treated with Fe_SA_@AH had lower BA than those with PBS and AH (Supplementary Fig. 27a). It is worth noting, however, that Fe_SA_@AH also decreased the BAce concentration (Supplementary Fig. 27b), indicating its dominant competitive role in ethanol elimination to endogenous ADH. Significant lower blood ALT and AST levels further confirmed the inflammation alleviation effect of Fe_SA_@AH on the liver (Fig. 5d). Additionally, administration of Fe_SA_@AH also significantly suppressed triglyceride and total cholesterol accumulation in ethanol-fed mice (Supplementary Fig. 28e–j).

The gut and its symbionts (the microbiota) are important, but usually overlooked, alcohol-metabolizing organs^44–46^. Chronic alcohol consumption caused histopathological changes in the colon, destroyed epithelial cells, atrophied goblet cells and resulted in inflammatory cell infiltration (Fig. 5e), and also weakened permeability (Fig. 5f), which may cause more microbial components to enter the bloodstream^47^. Alcohol also induced significant compositional shifts (β-diversity) in the gut microbiota of mice (Supplementary Fig. 29a), but showed limited effects on the Shannon index and percentage of Gram-negative bacteria (Supplementary Fig. 29b,c). Consistently^48^, the mean abundance of *Bacteroidota* increased in all alcohol-treated groups. Another dominant phylum, *Firmicutes*, decreased significantly in the PBS group compared with the blank group (Supplementary Fig. 29d). Interestingly, a significant loss of functional murine-mucoprotein-degrading bacteria, *Akkermansia* (*verrucomicrobiota*), and transitions of *Ileibacterium* and *Allobaculum* (blank) to *Bacteroides* and *Prevotellaceae_UCG-001* (PBS), were identified (Fig. 5g).

In terms of functional profiles, we found no significant intergroup gut microbial function changes due to ethanol-related processes (Supplementary Table 10). In accordance with previous research^47^, gut microbiota were determined to be indirectly involved in ethanol metabolism, especially acetate-induced microbial anaerobic respiration, such as the glycolysis/gluconeogenesis (ko00010) and pentose phosphate pathway (ko00030) (Supplementary Table 10). Alcohol consumption also induced significantly overexpressed pantothenate. Moreover, CoA biosynthesis (ko00770) and the citrate cycle (TCA) (ko00020) constituted important carbon unit donors for further processes (Fig. 5h), such as lipopolysaccharide (LPS) biosynthesis (ko00540)—LPS is widely recognized as an endotoxin that can induce hepatic inflammation^49^. This epithelial pathophysiological damage and intraluminal dysbiosis were significantly mitigated by Fe_SA_@AH compared with other AHs (Fig. 5e–h). Furthermore, as one of the final beneficial outputs, the concentration of blood LPS was significantly decreased in Fe_SA_@AH-treated mice (Fig. 5i).

In aggregate, we have demonstrated the design of a single-site iron-anchored amyloid hydrogel with remarkable catalytic oxidation capacity for alcohol as a highly efficient catalytic platform for in vivo alcohol metabolism. This work provides compelling evidence for the viability of a biomimetic-nanozyme-based hydrogel as an orally applied antidote for alcohol intoxication. Fe_SA_@AH demonstrates exceptional preference for acetic acid production, enabling a rapid decrease in blood alcohol levels while simultaneously mitigating the risk of excessive acetaldehyde accumulation, and markedly surpasses the effectiveness of existing alcohol intoxication antidotes that rely on a combination of natural enzymes. Unlike the predominantly liver-centric human intrinsic alcohol metabolism, orally administered Fe_SA_@AH directs this process towards the gastrointestinal tract, providing increased safety for the liver. In addition, despite this shift in the site of alcohol metabolism, there is no manifestation of additional adverse gastrointestinal symptoms; in fact, Fe_SA_@AH shows a remarkable alleviation of intestinal damage and dysbiosis induced by alcohol consumption, further demonstrating its potential for clinical translation.

The findings from our study outline a general and efficient strategy for synthesizing a diverse group of orally applied biomimetic nanozymes, and establish the foundation for future investigations aimed at maximizing the potential of artificial enzyme design in different therapeutic applications.

## Methods

### Synthesis of catalysts

BLG (>98%) was purchased from Davisco Foods International and purified using a previously reported protocol^50^. For a detailed description of BLG fibril preparation, see ref. ^51^. For the synthesis of Fe_SA_@FibBLG, 100 mg lyophilized BLG fibril powder was dispersed in a mixture of 8.0 ml ethanol and 1.9 ml PEG200. The dispersion was then subjected to argon bubbling for 30 min to remove the dissolved oxygen, followed by irradiation under a xenon lamp with an ultraviolet filter (250–380 nm, 27.9 mW cm^−2^, PLS-SXE300CUV) for 10 min to generate free radicals. Subsequently, 0.1 ml of 108.21 mg ml^−1^ Fe(NO_3_)_3_·9H_2_O EDTA solution was added dropwise to the dispersion of BLG fibrils under magnetic stirring for 12 h at 25 °C. Fe_SA_@BLG was prepared by the same synthesis procedure as for Fe_SA_@FibBLG, except that the BLG fibril powder was replaced by an equal amount of BLG powder. For the synthesis of FeNP@FibBLG, the as-obtained Fe_SA_@FibBLG dispersion was further ultraviolet-irradiated for 18 min under anaerobic conditions to reduce the iron ions. Finally, samples were collected by centrifugation at 4 °C, 11,100*g* for 10 min, washed by ethanol (10.0 ml × 6) and resuspended in 5.0 ml deionized water (pH 2). The powdered Fe_SA_@FibBLG, Fe_SA_@BLG and FeNP@FibBLG were obtained by lyophilization and stored at 4 °C.

### Characterizations

The high-resolution TEM images and elemental mappings were recorded with an FEI Talos F200X microscope at accelerating voltages of 80 kV and 200 kV, respectively. AFM images were obtained using a Bruker Multimode 8 scanning probe microscope. HAADF-STEM images were captured using an FEI Titan Themis G2 microscope equipped with a probe spherical aberration corrector and operated at 300 keV. The crystalline structure and phase purity were detected by a powder diffractometer (Siemens D500 with Cu Kα radiation (λ = 1.5406 Å)). The iron loadings on catalysts were analysed by inductively coupled plasma mass spectrometry (Elan DRC-e, Perkin Elmer). The X-ray absorption structure spectra (Fe K-edge) were collected at beamline BL44B2 of the SPring-8 synchrotron (Japan), operated at 8.0 GeV with a maximum current of 250 mA. Data were collected in transmission mode using a Si(111) double-crystal monochromator. The EXAFS data were analysed using the ATHENA module implemented in IFEFFIT software (CARS). XPS measurements were performed using a multipurpose spectrometer (Sigma Probe, Thermo VG Scientific) with a monochromatic Al Kα X-ray source. EPR spectra were acquired using a Bruker X-band (9.4 GHz) EMXplus 10/12 spectrometer equipped with an Oxford Instruments ESR-910 liquid helium cryostat. All spectra were collected under ambient conditions. Solution ^1^H NMR spectra were collected on a Bruker DRX 300 spectrometer (7.05 T; Larmor frequency, 300 MHz (^1^H)) in deuterated water (D_2_O) at room temperature.

### MD simulations

All of the AAMD simulations were performed on a GROMACS 2018 package using a gromacs54A force field^52^. The box size of the initial model was 12 × 12 × 30 nm^3^, including an SPC/E water model and 102 peptide chains (sequence, LACQCL)^19^ under three-dimensional periodic boundary conditions. A spherical cut-off of 1.0 nm was used for the summation of van der Waals interactions and short-range Coulomb interactions, and the particle-mesh Ewald method^53^. The temperature and pressure of the system were controlled by means of a velocity rescaling thermal thermostat and a Berendsen barostat. At first, the energy of the system was minimized in small steps to balance the initial velocity of the molecules. Then, the NPT ensemble using a leapfrog integrator with a time step of 1.0 fs was used to simulate the system for 8 ns at 300 K, which is sufficient for the balance of the system. Dynamic snapshot images were generated in Visual Molecular Dynamics 1.9.3^54^.

### DFT calculations

To investigate the interaction between iron ions and the system, one iron ion was inserted into the peptide dimer, and the structure was optimized by DFT using the CP2K software package^55^. The Perdew–Burke-Ernzerhof generalized gradient approximation functional was adopted to describe the electronic exchange and correlation, in conjunction with the DZVP-MOLOPT-SR-GTH basis set for all atoms (C, H, O, N, Fe). The structure was optimized with the spin multiplicity to treat the doublet spin state and the charge of the iron ion was set to +2*e*. The convergence criterion for the absolute value of the maximum force was set to 4.5 × 10^−4^ a.u. and the r.m.s. of all forces to 3 × 10^−4^ a.u. Grimme’s DFT-D3 method was adopted for correcting van der Waals interactions^56^.The interaction of the system was characterized by the independent gradient model method, and the based isosurface maps were rendered by Visual Molecular Dynamics from the cube files exported from Multiwfn 3.8 (ref. ^57^).

### Peroxidase-like activity

The peroxidase-like activities of nanozymes were assessed at 37 °C using 350 μl of HAc–NaAc buffer (0.1 M, pH 4.0) with varied nanozyme concentrations, using TMB as the substrate. Following the addition of 20 μl of TMB solution (20 mM in dimethylsulfoxide) and 20 μl of H_2_O_2_ solution (2 M), 10 μl of nanozymes with varying concentrations was introduced into the system. The catalytic oxidation of TMB (oxTMB) was quantified by measuring the absorbance at 652 nm via an ultraviolet–visible spectrometer. The steady-state kinetics analysis was executed by modifying the concentrations of TMB and H_2_O_2_. To derive the Michaelis–Menten constant, we performed Lineweaver–Burk plot analysis using the double reciprocal of the Michaelis–Menten equation, *ν* = *ν*_max_ × [*S*]/(*K*_m_ + [S]), where ν denotes the initial velocity, *ν*_max_ represents the maximum reaction velocity, [*S*] indicates the substrate concentration and *K*_m_ is the Michaelis constant. Additionally, the catalytic rate constant (*k*_cat_) was computed as *k*_cat_ = *ν*_max_/[*E*], where [*E*] signifies the molar concentration of metal within the nanozymes. By employing diverse pH buffer solutions, we explored the pH dependency of the peroxidase-like activity of nanozymes, spanning a range from pH 2 to 9. Similarly, we investigated its temperature sensitivity by observing its activity at various temperatures, progressively increasing from 20 °C to 60 °C.

### Catalytic oxidation activity on alcohol and acetaldehyde

The catalytic oxidation activities of nanozymes on both alcohol and acetaldehyde were carried out at 37 °C in 350 μl of HAc–NaAc buffer (0.1 M, pH 4.0), with varying nanozyme concentrations (10 μl). Subsequent to adding 20 μl of H_2_O_2_ solution (2 M), 20 μl of ethanol or acetaldehyde solution (2 mM) was introduced into separate tubes containing the reaction mixture. Quantification of the catalytic oxidation of ethanol or acetaldehyde was performed using the Ethanol Assay Kit (ab65343) and Acetaldehyde Assay Kit (ab308327) from Abcam Biotechnology. Through altering the concentrations of ethanol or acetaldehyde, steady-state kinetics analysis was carried out, and the Michaelis–Menten constant was determined by analysing Lineweaver–Burk plots involving the double reciprocal of the Michaelis–Menten equation. Additionally, the identification of the reaction products was confirmed by ^1^H NMR spectrometry.

### Catalytic activity assessment of nanozymes during in vitro simulation of the digestion process

We adhered to the INFOGEST standard protocol for nanozyme digestion to replicate the physiological human gastrointestinal digestion process^58^. In this methodology, stock solutions of simulated gastric fluid and simulated intestinal fluid were prepared and equilibrated at 37 °C prior to use. For gastric digestion, 2 ml of the nanozyme (1 mg ml^−1^) was mixed with 2 ml of simulated gastric fluid stock solution, and porcine pepsin solution was added to achieve a final enzyme activity of 500 U per mg of protein. CaCl_2_(H_2_O)_2_ was then introduced into the mixture to reach a final concentration of 0.15 mM prior to adjusting the pH to 3 using 5 M HCl. The mixture was transferred to a water bath shaker (VWR 462-0493) at 37 °C and sampled at 30 and 60 min, after which NaOH solution was used to deactivate the enzyme. Following the gastric digestion, pancreatin (0.1 mg ml^−1^) was dissolved in simulated intestinal fluid containing 0.6 mM CaCl_2_ and added to the gastric digests in a 1:1 (v/v) ratio to initiate intestinal digestion, which lasted for 120 min at 37 °C with regular sampling every 30 min. The samples were freeze-dried immediately after collection for enzyme activity evaluation experiments using TMB as a substrate, in which the amount of nanozyme after digestion was normalized.

### Hydrogel formation

Gelation of Fe_SA_@FibBLG dispersion containing AuNPs (Fe_SA_@AH) was achieved following our previously reported procedure with some modifications^40^. For the synthesis of AuNPs, all glassware was cleaned with freshly prepared aqua regia (HCl:HNO_3_ = 3:1 vol/vol) and then thoroughly rinsed with water. A 2 ml solution of BLG fibrils (2.0 wt%, pH 2.0) was mixed with a 40 mM HAuCl_4_ solution to reach a final protein:gold mass ratio of 14.7:1. The mixture underwent a chemical reduction through the dropwise addition of a NaBH_4_ solution (0.8 ml) under a nitrogen atmosphere. The resulting solution was then dialysed to remove any remaining NaBH_4_ and concentrated to 2 ml with a dialysis membrane (Spectra/Por, molecular weight cut-off, 6–8 kDa, Spectrum Laboratories) against a 6 wt% PEG solution (*M*_r_ ≈ 35,000, Sigma-Aldrich) at pH 2.0. TEM imaging of AuNPs stabilized by BLG fibrils revealed three-dimensional particles with an average size of 1.32 nm (Supplementary Fig. 21a), determined by analysing six TEM images using ImageJ software v.1.8.0. For the preparation of Fe_SA_@AH, 2 g of Fe_SA_@FibBLG powder was dissolved in the resulting AuNP-attached BLG fibril solution (2 ml). The mixture was then transferred into a plastic syringe, the top part of which had been previously cut. The plastic syringe was covered with a section of a dialysis tube (Spectra/Por, molecular weight cut-off, 6–8 kDa), and the head of the syringe was positioned in direct contact with an excess of 300 mM NaCl solution at pH 7.4 for at least 16 h in a 4 °C cold room to facilitate gelation. The resulting hydrogel sample was kept under 4 °C. The working hydrogel was freshly prepared by mixing the aforementioned hydrogel with 0.1 ml of a glucose solution (8.0 M) immediately before further characterization or detoxification use. A BLG fibril hydrogel was obtained using the same procedure, except that the Fe_SA_@FibBLG was replaced with an equal amount of BLG fibril dispersion.

### Murine models

Male wild-type C57BL/6 mice, 20–25 g and 8–10 weeks old, were purchased from Beijing Vital River Laboratory Animal Technology. All of the murine experiments in the current study were approved by the Regulations of Beijing Laboratory Animal Management (approval number AW40803202-5-1) and conducted according to the guidelines set forth in the Institutional Animal Care and Use Committee of China Agricultural University.

### Acute model

Thirty-two male C57BL/6 mice were randomly divided into four groups after 12 h fasting. Mice were orally gavaged with AH and Fe_SA_@AH (at doses of 10 ml per kg (body weight)), and two groups of mice received the same volume of PBS (as controls, the blank and the PBS groups), respectively. After 20 min of adaptation, mice from the AH, Fe_SA_@AH, and PBS groups were orally administered an alcohol liquid diet (10 g per kg (body weight)), while the same volume of PBS was administered for the blank group. All the mice were killed 6 h later.

### Chronic model

A mouse model of chronic and binge ethanol feeding (NIAAA model) was conducted following the protocol proposed by Bertola et al.^43^. In brief, after 5 days of ad libitum Lieber–DeCarli diet adaptation, 32 mice were randomly divided into four groups: (1) a control group (Con) of mice were pair-fed with the control diet; (2) an ethanol diet group (EtOH); (3) an ethanol diet group with additional 10 ml per kg (body weight) AH; and (4) an ethanol diet group with additional 10 ml per kg (body weight) Fe_SA_@AH. The ethanol-fed groups were granted unrestricted access to the ethanol Lieber–DeCarli diet containing 5% (vol/vol) ethanol for 10 days, and additionally received daily morning (9:00) gavage of PBS, AH or Fe_SA_@AH, respectively. The control group was pair-fed with an isocaloric control diet and daily control-liquid gavage. All animals were maintained in specific pathogen-free conditions, at a temperature of 23 ± 1 °C and 50–60% humidity, under a 12 h light/dark cycle, with access to autoclaved water. On day 16, both the ethanol-fed and pair-fed mice were orally administered a single dose of ethanol (5 g per kg (body weight)) or isocaloric maltose dextrin at 9:20, respectively, and killed 6 h later. The body weight of mice was recorded every 2 days.

### MicroPET

After overnight fasting, mice were gavaged with 0.1 ml [^18^F]FDG-labelled Fe_SA_@AH. Then, mice were anaesthetized with oxygen containing 2% isoflurane, and placed in and fixed in a prone position in an imaging chamber. Time-series images were obtained with an Inveon microPET/CT scanner (Siemens); the scanner parameters were a 15 min CT scan (80 kVp, 500 μA, 1,100 ms exposure time) followed by a 10 min PET acquisition. Quantification of images was performed by AMIDE software 3.0.

### Alcohol tolerance test

Approximately 10 µl of blood was collected from the submandibular vein at 30, 60, 90, 120, 180 and 300 min after alcohol exposure. In the chronic model, sampling was conducted after the binge ethanol feeding. Blood alcohol concentration (BAC) was determined using a test kit from Abcam Biotechnology (ab65343). BACs were normalized to mice body weights as previously described^8^. Normalized BAC, BAC_nor_, was calculated using the equation: BAC_nor_ = BAC_i_ × (BWT_i_/BWT_ave_), where BAC_i_ and BWT_i_ denote the blood alcohol level and body weight of mice, respectively, and BWT_ave_ represents the average weight of all mice in each set of experiments. The quantification of the BAce concentration was carried out using a test kit obtained from Abcam Biotechnology (ab308327), and the normalization process was conducted using the same method as for the BAC.

Alcohol tolerance time was the duration between alcohol administration and the absence of righting reflex, while the duration of the absence of righting reflex was recorded as the sobering-up time. Mice that became ataxic were considered to have lost their righting reflex, and were then placed face up. The time point at which the mice returned to their normal upright position signified they had regained their righting reflex.

### MWM test

An MWM test^59^ was conducted by Anhui Zhenghua Biologic Apparatus Facilities, as described previously. Specifically, the MWM apparatus comprised a large circular pool (120 cm diameter and 40 cm height) which was filled with TiO_2_-dyed, 25 °C thermostatic water, and a 10-cm-diameter platform was positioned and fixed 2 cm below the water surface. Before acute ethanol exposure, mice received four rounds of daily training for 6 days. Each trial was limited to 60 s, and the time that it took for the mice to successfully locate the platform was recorded. On day 7, mice were retested (no platform condition) 5 h after ethanol feeding (the time point by which all mice regained their consciousness and mobility). The tested items included trajectory, path length, escape latency and swimming speed (MWM animal behaviour video tracking system, Morris v.2.0).

### Biochemical assays

Blood samples were collected through cardiac puncture from anaesthetized mice 6 h after alcohol gavage. Prior to testing, samples were maintained at ambient temperature for 4 h, and then centrifuged (864.9*g*, 4 °C) for 20 min. Supernatants were suctioned and stored at −80 °C for further analysis. Serum ALT, AST, triglycerides and total cholesterol were measured by a Hitachi Biochemistry Analyzer 7120 (Hitachi High-Tech).

Weighed liver tissues were collected and immersed immediately in 10% neutral buffered formaldehyde. After overnight fixation, tissues were embedded in paraffin and cut into 5 μm sections for further haematoxylin and eosin (H&E) and oil red O (Sigma) staining. Images were captured by a Nikon Eclipse TI-SR fluorescence microscope. Fresh liver was homogenized in chilled normal saline and centrifuged (1,500*g*, 4 °C) for 15 min. GSH and MDA levels of the resultant supernatant were detected using the GSH assay kit (ab65322) and the lipid peroxidation (MDA) assay kit (ab118970), respectively. Hepatic and cellular lipid content was isolated using the chloroform/methanol-based method^60^, and quantified by using the triglyceride assay kit (ab65336) and the mouse total cholesterol ELISA kit (ab285242, SSUF-C), respectively.

### Colon histology and immunohistochemistry

Colon length, caecum to anus, was measured, and the distal colon was washed with saline, with one-half being fixed with 4% paraformaldehyde, and the other half stored at −80 °C. Histological measurements of the colon were the same as those for the liver.

For immunofluorescence, colon tissues were treated with EDTA buffer and boiled to expose the antigens. Tissues were then incubated overnight at 4 °C with primary antibody and washed three times for 5 min each with PBS. Subsequently, colon tissues were covered with secondary antibody and incubated at room temperature in the dark for 50 min, followed by another set of three 5 min washes with PBS. The resultant sections were mounted with a mounting medium and stained with 4,6-diamidino-2-phenylindole. Slides were then covered, and the images were captured using a Nikon Eclipse Ti inverted fluorescence microscope.

### Microbiota changes

Faecal samples were collected within 5 min after defecation into a sterile tube and stored at −80 °C. Microbial genome DNA was extracted from faeces by using the DNeasy PowerSoil Pro Kit (QIAGEN) according to the manufacturer’s instructions, and the variable 3–4 (V4-v4) region of the 16S rRNA gene was PCR-amplified using barcoded 338F-806R primers (forward primer, 5′-ACTCCTACGGGAGGCAGCAG-3′; reverse primer, 5′-GGACTACHVGGGTWTCTAAT-3′). PCR components contained 25 μl of Phusion High-Fidelity PCR Master Mix, 3 μl (10 μM) of each forward and reverse primer, 10 μl of the DNA template, 3 μl of DMSO and 6 μl of double-distilled H2O. The following cycling conditions were used: initial denaturation at 98 °C for 30 s, followed by 25 cycles of denaturation at 98 °C for 15 s, annealing at 58 °C for 15 s, and extension at 72 °C for 15 s, and a final extension of 1 min at 72 °C. PCR amplicons were purified using Agencourt AMPure XP Beads (Beckman Coulter) and quantified using a PicoGreen dsDNA Assay Kit (Invitrogen). After quantification, amplicons were pooled in equal amounts, and 2 × 150 bp paired-end sequencing was performed using the Illumina Miseq PE300 platform at GUHE Info Technology. Amplicon sequence variants (ASVs) were denoised and clustered by the UNOISE algorithm. Taxa bar plots, and α- and β-diversity analysis, were performed with QIIME 2 v.2020.6 and the R package v.3.6.3. Metabolic function was predicted using PICRUSt2, and the output file was further analysed using the STAMP software package (v.2.1.3).

### Reporting summary

Further information on research design is available in the Nature Portfolio Reporting Summary linked to this article.

## Online content

Any methods, additional references, Nature Portfolio reporting summaries, source data, extended data, supplementary information, acknowledgements, peer review information; details of author contributions and competing interests; and statements of data and code availability are available at 10.1038/s41565-024-01657-7.

### Supplementary information

Supplementary Information Supplementary Methods, Discussion, Figs. 1–29, Tables 1–10, Gating strategy for flow cytometry and References.

Reporting Summary

### Source data

Source Data Fig. 1 Source data for Fig. 1.

Source Data Fig. 2 Source data for Fig. 2.

Source Data Fig. 3 Source data for Fig. 3.

Source Data Fig. 4 Source data for Fig. 4.

Source Data Fig. 5 Source data for Fig. 5.

## Data Availability

All the data that validates the outcomes of this study are included in the Article and its Supplementary Information files. For any other relevant source data, interested parties can obtain them from the corresponding authors upon reasonable request. Source data are provided with this paper.

## Code availability

Simulation files and code used for modelling iron-anchored BLG fibril segments can be accessed via Zenodo at: 10.5281/zenodo.10819612 (ref. ^61^).
