# Supplementary material for: Single-site iron-anchored amyloid hydrogels as catalytic platforms for alcohol detoxification
Source: Nat Nanotechnol. 2024 May 13;19(8):1168–77. doi: 10.1038/s41565-024-01657-7 (PMC11329373; doi:10.1038/s41565-024-01657-7)
Supplement: Supplementary file 1 — Supplementary Methods, Discussion, Figs. 1–29, Tables 1–10, Gating strategy for flow cytometry and References. [file 41565_2024_1657_MOESM1_ESM.pdf]

nature nanotechnology

Article https://doi.org/10.1038/s41565-024-01657-7

Single-site iron-anchored amyloid hydrogels as catalytic platforms for alcohol detoxification

In the format provided by the authors and unedited

Table of Contents:

**Supplementary Method**	Page 3
**Supplementary Discussion**	Page 4
**Supplementary Fig. 1**	Page 5
**Supplementary Fig. 2**	Page 6
**Supplementary Fig. 3**	Page 7
**Supplementary Table 1**	Page 8
**Supplementary Fig. 4**	Page 9
**Supplementary Fig. 5**	Page 9
**Supplementary Fig. 6**	Page 10
**Supplementary Fig. 7**	Page 11
**Supplementary Fig. 8**	Page 12
**Supplementary Fig. 9**	Page 13
**Supplementary Fig. 10**	Page 14
**Supplementary Table 2**	Page 15
**Supplementary Fig. 11**	Page 16
**Supplementary Fig. 12**	Page 16
**Supplementary Fig. 13**	Page 17
**Supplementary Table 3**	Page 17
**Supplementary Fig. 14**	Page 18
**Supplementary Table 4**	Page 18
**Supplementary Fig. 15**	Page 19
**Supplementary Table 5**	Page 19
**Supplementary Fig. 16**	Page 20

**Supplementary Table 6**	Page 20
**Supplementary Fig. 17**	Page 21
**Supplementary Fig. 18**	Page 22
**Supplementary Fig. 19**	Page 23
**Supplementary Fig. 20**	Page 24
**Supplementary Fig. 21**	Page 25
**Supplementary Fig. 22**	Page 26
**Supplementary Fig. 23**	Page 27
**Supplementary Fig. 24**	Page 28
**Supplementary Fig. 25**	Page 29
**Supplementary Fig. 26**	Page 30
**Supplementary Fig. 27**	Page 31
**Supplementary Fig. 28**	Page 32
**Supplementary Fig. 29**	Page 33
**Supplementary Table 7**	Page 34
**Supplementary Table 8**	Page 35
**Supplementary Table 9**	Page 36
**Supplementary Table 10**	Page 37
**Gating strategy for flow cytometry**	Page 43
**References**	Page 44

Supplementary Method

In vitro toxicity assessment

Cell viability assays. The human colorectal cancer cells (Caco-2, HTB-37) were obtained from the Cell Bank of the Chinese Academy of Sciences (Shanghai, China). Six-well plate-seeded Caco-2 cells (2 mL and 3×10^5 cells/well) were treated with AH or Fe_{SA}@AH after 24 h of resuscitation under the following conditions: DMEM containing 10%(v/v) fetal bovine serum (FBS), nonessential amino acids, and 1% (v/v) penicillin–streptomycin (10,000 U/mL penicillin and 10 mg/mL streptomycin) at 37 °C in 5% CO_2. The same amounts (4.0 g/mL, 40 μL) of AH or Fe_{SA}@AH were incubated with cells for another 24 h at 37 °C in 5% CO_2. Cells treated with PBS were used as a negative control. After exposure, cells were washed three times in cold PBS, centrifuged at 216 × g for 5 min, and re-suspended in 500 μL of binding buffer. The cells were then Annexin V-FITC (Invitrogen™) stained, and the cellular fluorescence was detected by using a flow cytometer (FACSCalibur 2, U.S.A.) equipped with a 488 nm excitation wavelength argon ion laser. Green fluorescence and red fluorescence were measured above 530/30 nm (FL1) and 670 nm (FL3), respectively.

***In vivo* safety evaluation.** Male C57BL/6 mice (n=6) were hypodermically injected with our nanozymes to assess their allergenicity and toxicity. Mouse were anesthetized by intramuscular injections of ketamine (85 mg kg^{-1} BW) and xylazine (10 mg kg^{-1} BW) on posterior thighs. After removing their back hair, mice were subcutaneously injected with 50 µl of AH or Fe_{SA}@AH (1 mg/mL) at time point 0 and 24 h, respectively. Mice injected with H_2O_2 solution or PBS buffer were set as positive and negative controls, respectively. After the skin test, mice were sacrificed, and skin tissue was collected and fixed for further analysis. Skin samples were stained with hematoxylin and eosin (H&E), and the apoptotic cells were detected by terminal deoxynucleotidyl transferase-mediated dUTP nick end labeling (TUNEL, Roche Molecular Biochemicals). Images were captured by Zeiss Axio Vert AI or Zeiss LSM980 (ZEN 2012) confocal microscopes, respectively, and processed with CaseViewer 3.3 software.

Statistics and reproducibility

For physicochemical experiments in this study, quantitative data were presented as the mean ± s.d. All experiments were performed with at least three independent measurements, while the images shown are from six technical replicates (n = 6). For biological studies, data are shown in the form of mean ± SEM. All experiments were performed with at least three biological replicates. Statistical analyses were performed using one-way analysis of variance (ANOVA) with the Tukey–Kramer test, and the significance levels are $*P < 0.05$, $**P < 0.01$, $***P < 0.001$and $****P < 0.0001$. No statistical methods were used to pre-determine sample sizes, but our sample sizes are similar to those reported in previous publications[1]. Data distribution was assumed to be normal, but this was not formally tested. Data collection and analysis were performed blind to the conditions of the experiments. No animal or data points were excluded from the analysis for any reason. Data analysis was done using Origin 2021 and GraphPad Prism 9.0 software unless otherwise stated.

Supplementary Discussion

XPS analysis

X-ray photoelectron spectroscopy (XPS) was utilized to determine the binding states of carbon (C), nitrogen (N), oxygen (O) and iron (Fe) in Fe_{SA}@FibBLG (Supplementary Fig. 4). Unlike the XPS spectrum of FibBLG support, which exhibited no signal within the Fe 2p range, the Fe 2p XPS spectrum of Fe_{SA}@FibBLG was deconvoluted into six peaks, validating the presence of Fe species within the BLG fibril framework (Supplementary Fig. 4b and 5b). Peaks at 724.2 eV and 710.8 eV corresponded to the 2p3/2 and 2p1/2 states of Fe^{2+}, respectively. Meanwhile, peaks at 728 eV and 712 eV were indicative of the 2p3/2 and 2p1/2 states of Fe^{3+}, respectively. Furthermore, peaks at 718.3 eV and 732.2 eV corresponded to the satellite peaks of Fe^{2+}.[2]. Analysis of the XPS data revealed that approximately 62% of the single-site Fe in Fe_{SA}@FibBLG was present in the Fe^{2+} state. The existence of Fe^{2+} was ascribed to the reducing effect of BLG fibrils, as verified by our previous report[3]. Deconvolution of the C1s XPS spectrum for Fe_{SA}@FibBLG revealed three peaks, with binding energies at 288.2 **(**C=O**)**, 286.2 **(**C-O/C-N**)**, and 284.8 eV **(**C-C/C-H**)**, respectively, derived from the BLG fibril support (Supplementary Fig. 4c)[4,5]. Noteworthy in the N 1s spectrum of FibBLG are two distinct peaks at binding energies of 400.58 eV (amide N) and 399.87 eV (N-Hx) (Supplementary Fig. 5d). An additional peak emerged at a binding energy of 398.98 eV (Fe-N) in the N 1s XPS spectrum of Fe_{SA}@FibBLG, accompanied by a substantial change in the ratio between amide N and N-Hx (Supplementary Fig. 4d), revealing the formation of Fe-N coordination. The peak shape and position in the O 1s spectrum remain nearly identical before and after iron loading (Supplementary Figs. 4e and 5e), providing further evidence for the prevalence of Fe-N coordination over Fe-O coordination.

a

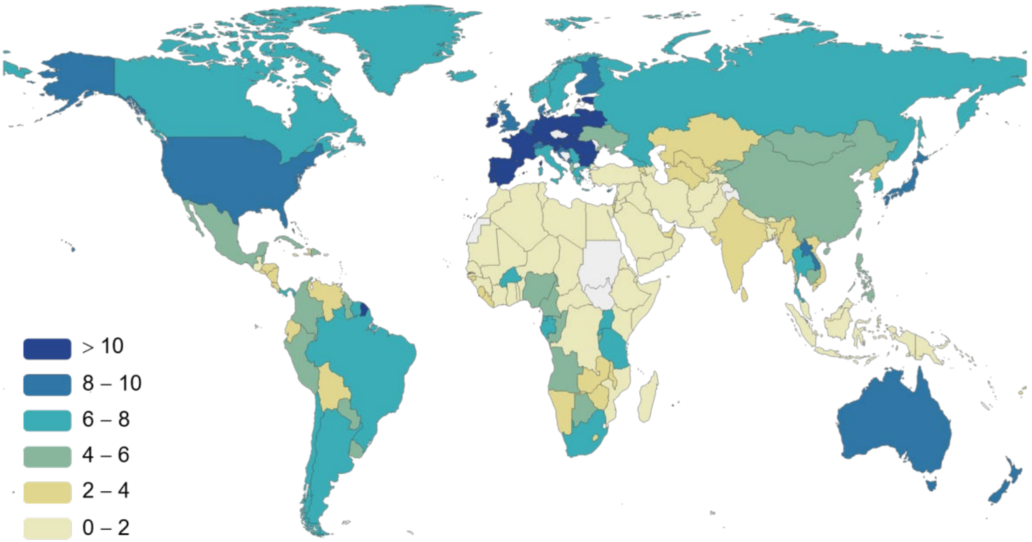

Supplementary Fig. 1. Average consumption of pure alcohol in liters per year among all adults aged 15+ in 2019 (Data source: World Health Organization).

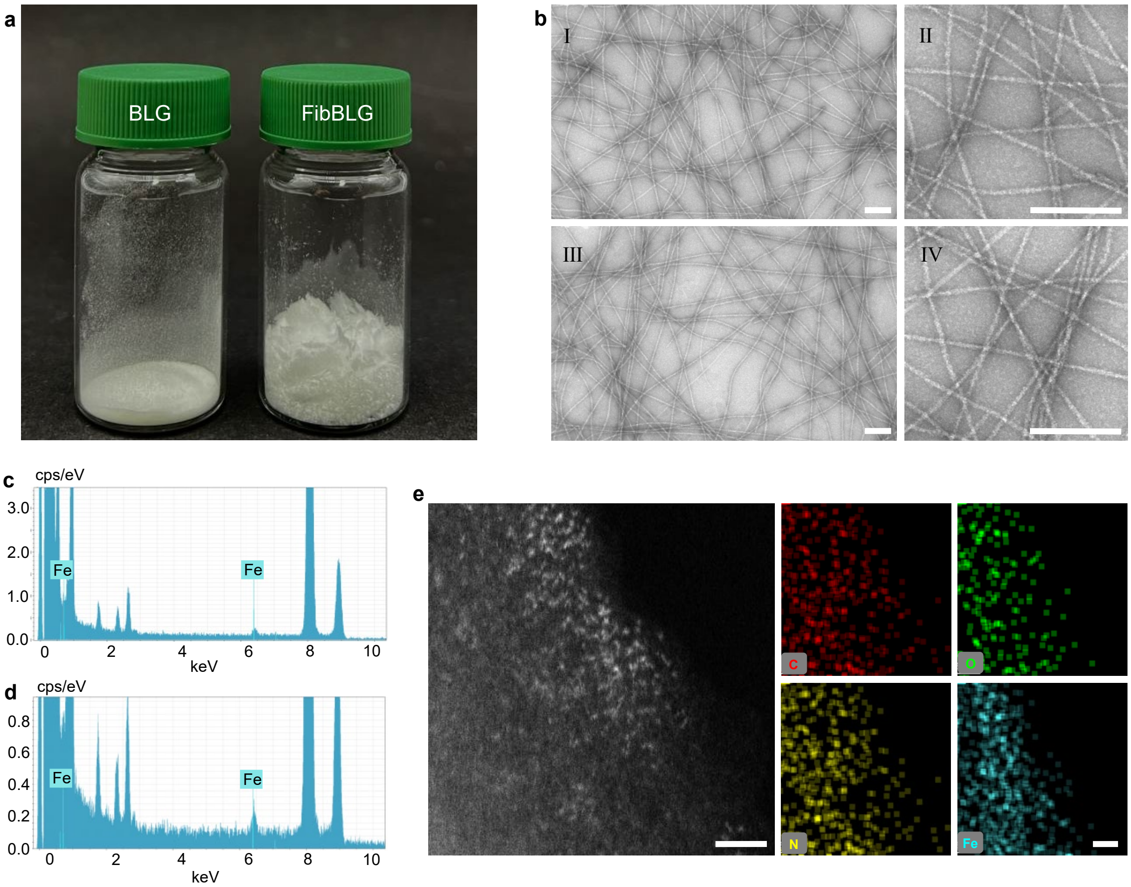

Supplementary Fig. 2. a, Visual observation 0.12 g native BLG and FibBLG. b, TEM images of Fe_{SA}@FibBLG (I and II) and FibBLG (III and IV). scale bars, 200 nm. c, EDX elemental mapping analysis of Fe_{SA}@FibBLG of the region in Fig. 1d. d,e, Enlarged HAADF-STEM (d) and the corresponding intensity map (e) of Fe_{SA}@FibBLG. e, EDX elemental mapping analysis of Fe_{SA}@FibBLG in (e). Scale bar, 2 nm. Images in b and e illustrate results consistent across six technical replicates (n=6), demonstrating reproducible outcomes.

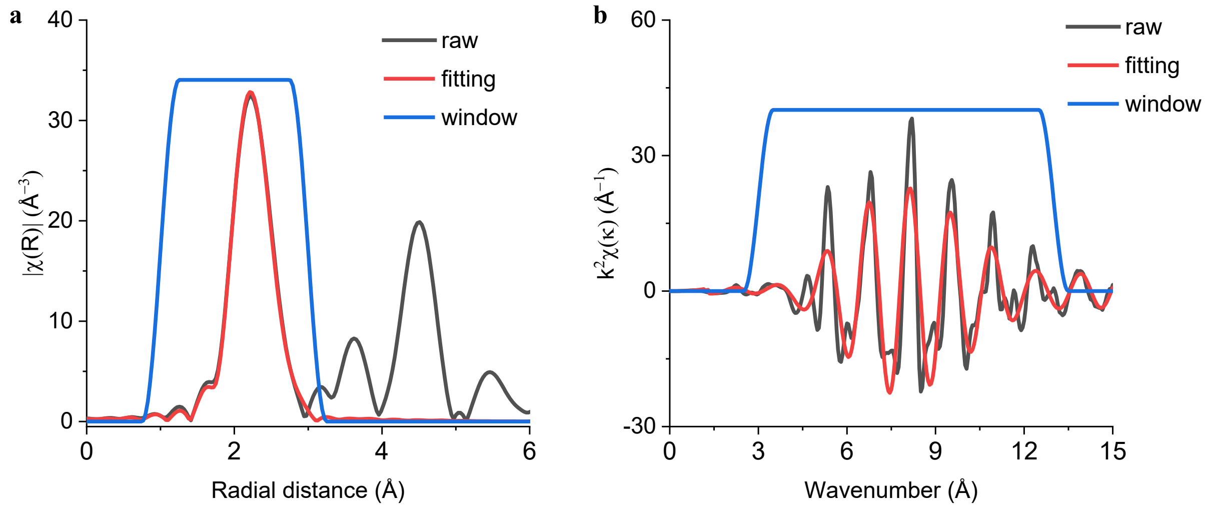

Supplementary Fig. 3. EXAFS fitting for Fe foil. a, K-space with raw (red) and fitted (grey) data. Window (blue) 3.0-12.5 $Å^{-1}$, k-weight = 2, Hanning window, dk = 1; b, R-space with raw (red) and fitted (grey) data. Window (blue) 1-3. Å, k-weight = 2, Hanning window, dk = 0.5. Fitting results are summarized in **Supplementary Table 1**.

Supplementary Table 1. EXAFS fitting parameters at the Fe K-edge for various samples.

Sample	Shell	CN[a]	R(Å)[b]	σ2 ($Å^2 \cdot 10^{-3}$)[c]	ΔE_0(eV)[d]	R factor (%)
Fe Foil	Fe-Fe_{SA}	8*	2.47 ± 0.01	0.0051	5.67 ± 2.12	0.7
	Fe-Fe_2	6*	2.85 ± 0.02	0.0058	5.24 ± 3.46	
Fe_{SA}@FibBLG	Fe-N	4.5 ± 0.3	1.94 ± 0.02	0.0064	8.46 ± 1.91	0.3

[a]*CN*, coordination number; [b]*R*, distance between absorber and backscatter atoms; [c]σ^2, Debye-Waller factor to account for both thermal and structural disorders; [d]ΔE_0, inner potential correction; *R* factor indicates the goodness of the fit. $S0^2$ was fixed to 0.82 according to the experimental EXAFS fit of Fe foil by fixing CN as the known crystallographic value. Fitting range: $3.0 \leq k$ (/Å) ≤ 13 and $1.0 \leq R$ (Å) $\leq$ ~3 (Fe foil); $3.0 \leq k$ (/Å) ≤ 11.0 and $1.0 \leq R$ (Å) $\leq$ ~2.5(Fe_{SA}@FibBLG).

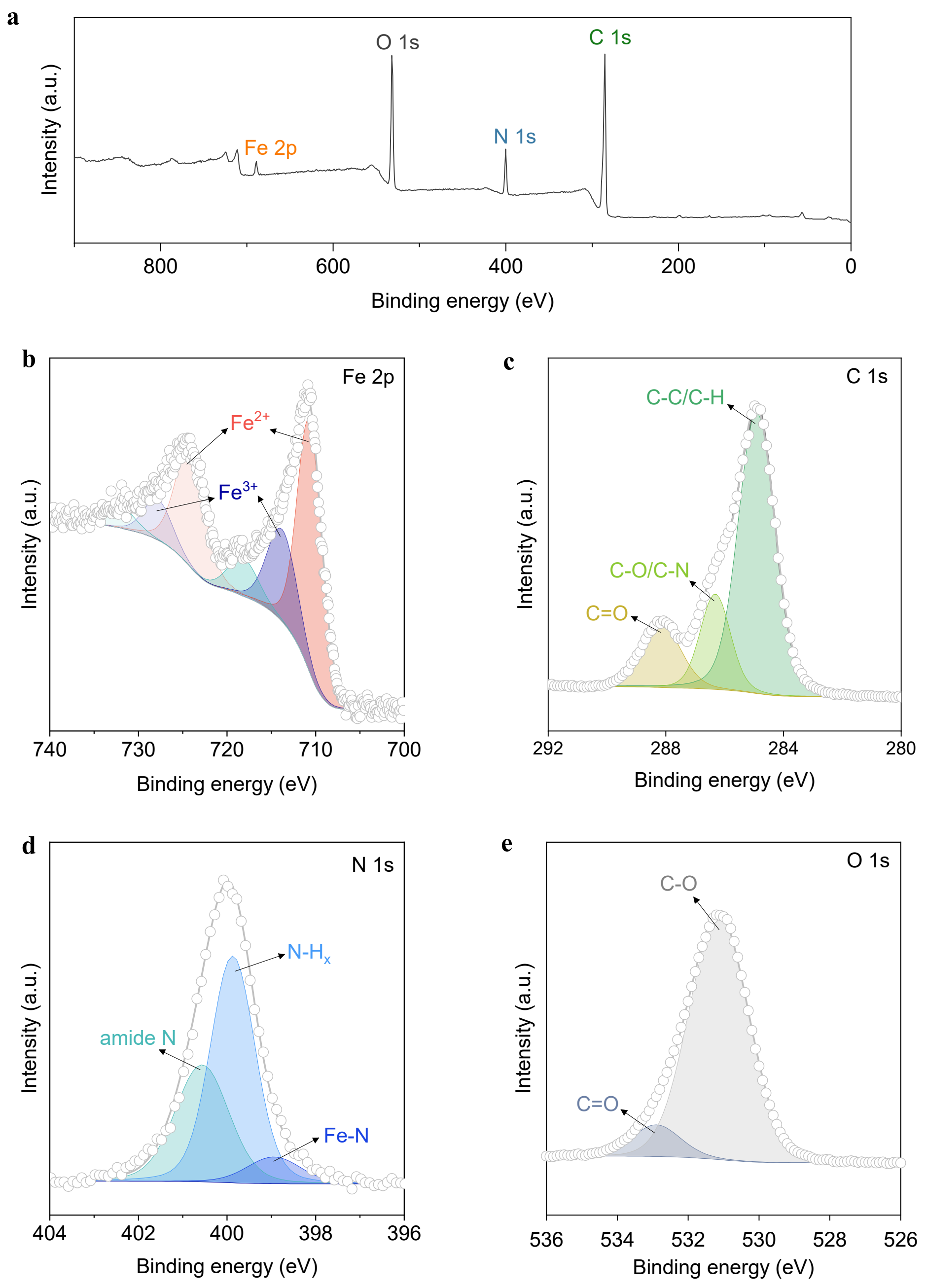

Supplementary Fig. 4. XPS survey (a) and the deconvoluted C 1s (b), N 1s (c), O 1s (d), and Fe 2p (e) spectra of Fe_{SA}@FibBLG.

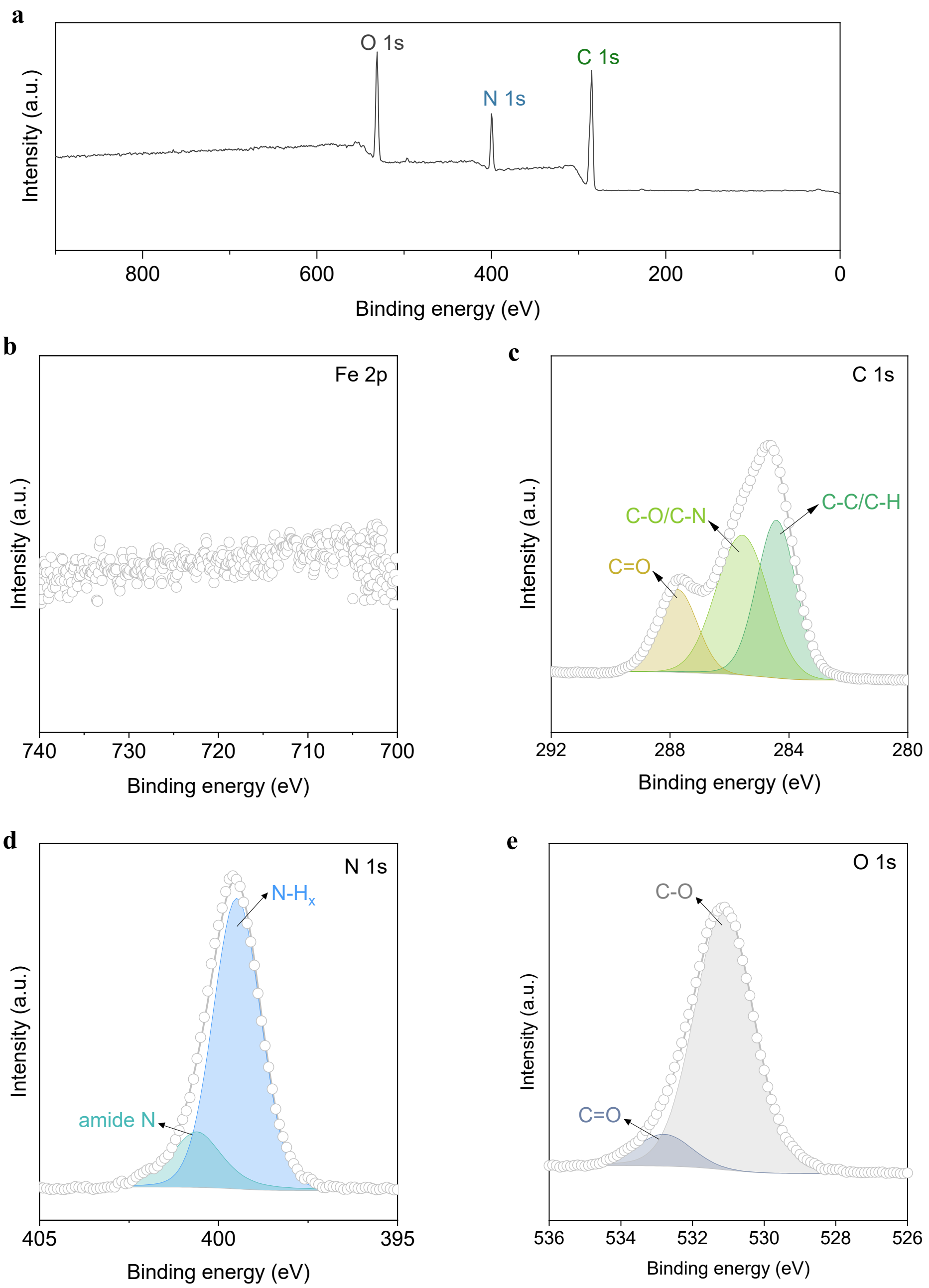

Supplementary Fig. 5. XPS survey (a) and the deconvoluted C 1s (b), N 1s (c), O 1s (d), and Fe 2p (e) spectra of FibBLG.

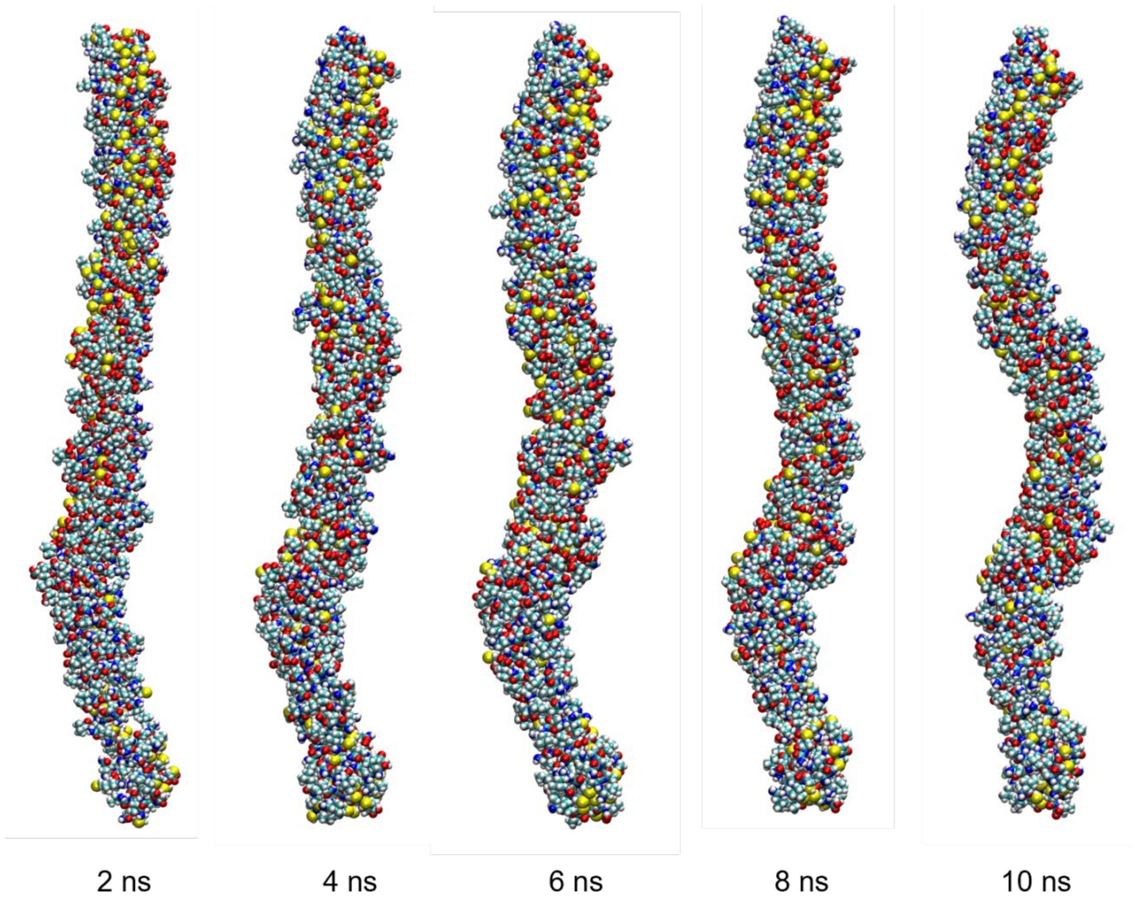

Supplementary Fig. 6. Representative snapshots of the structure of peptide assembly in the process of GROMACS using gromacs54A force field.

Supplementary Fig. 7. The typical interpretation of the color-coding method for representing the sign(λ_2)ρ function in IGM and IGMH maps.[6]

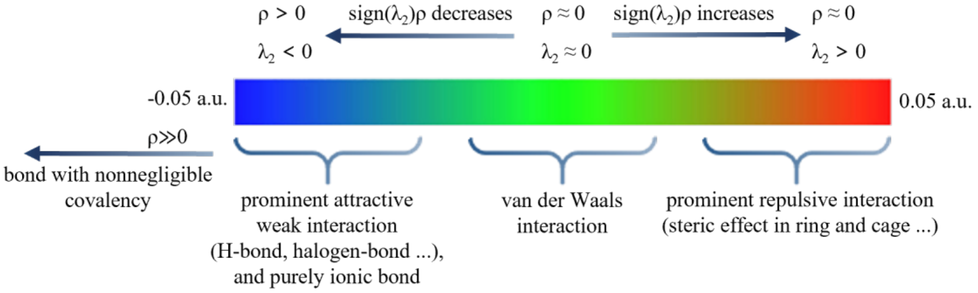

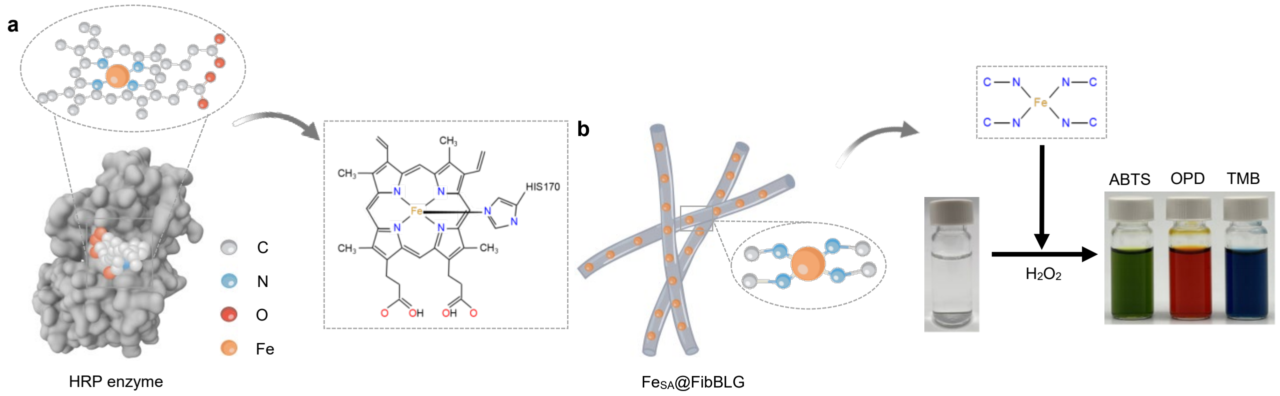

Supplementary Fig. 8. a,b, Model diagram and chemical environment of the Fe active site in horseradish peroxidase (HRP) enzyme and Fe$_{SA}$@FibBLG. The findings validate that the Fe active site in Fe$_{SA}$@FibBLG, synthesized in this study, closely resembles the structure observed in the natural horseradish peroxidase (HRP) enzyme. c, Peroxidase mimicking of Fe$_{SA}$@FibBLG by catalyzing the oxidation of ABTS, OPD, and TMB.

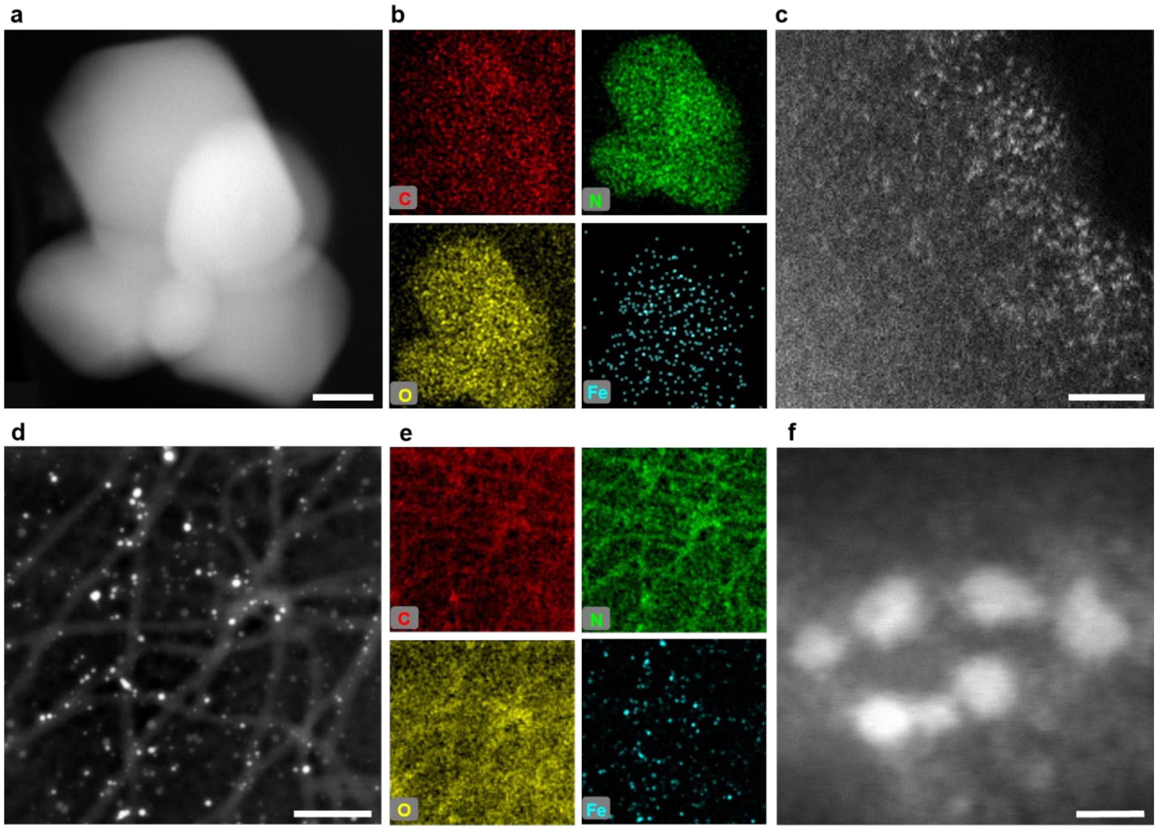

Supplementary Fig. 9. a, b, HAADF-STEM image (a) and the corresponding EDS mapping images (C, red; N, green; O, yellow; Fe, blue; b) of Fe_{SA}@BLG (scale bar, 200 nm). c, Enlarged HAADF-STEM image of Fe_{SA}@BLG (scale bar, 2 nm). d, e, HAADF-STEM image (d) and the corresponding EDS mapping images (e, red; N, green; O, yellow; Fe, blue; b) of FeNP@FibBLG (scale bar, 200 nm). f, Enlarged HAADF-STEM image of FeNP@FibBLG (scale bar, 10 nm). Images illustrate results consistent across six technical replicates (n=6), demonstrating reproducible outcomes.

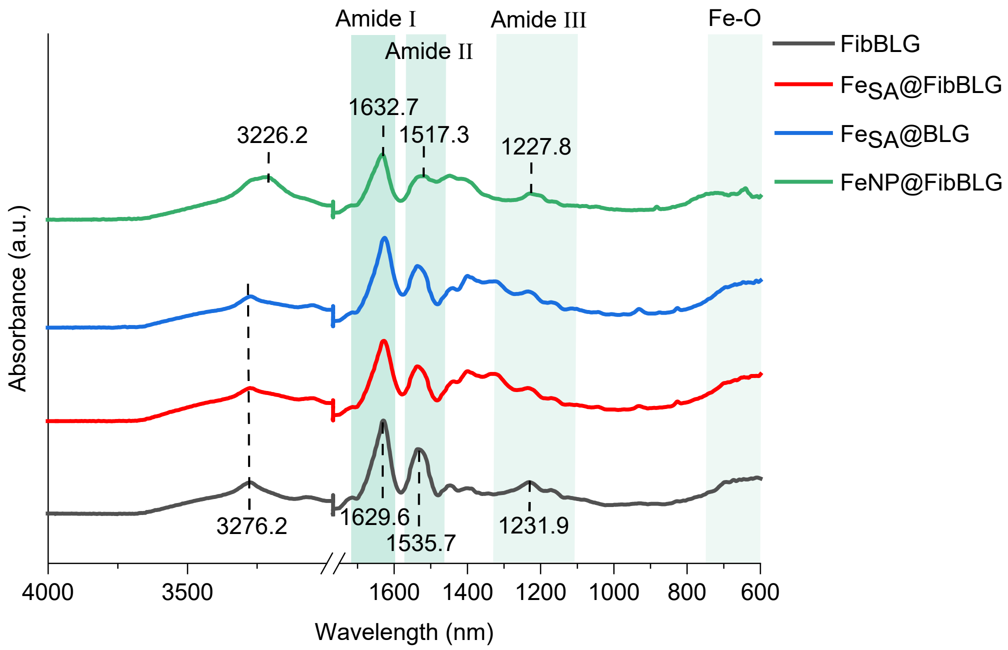

Supplementary Fig. 10. FTIR spectra of FibBLG, Fe_{SA}@FibBLG, Fe_{SA}@BLG, and FeNP@FibBLG.

Supplementary Table 2. Mass percentage of Fe in nanozymes.

Sample name	Metal loading (wt%, Fe)	SD (%)
Fe_{SA}@FibBLG	1.21	0.0218
Fe_{SA}@BLG	1.09	0.0304
FeNP@FibBLG	1.48	0.0209

The content is determined by ICP-MS.

Note: Here, we would like to perform a concise calculation regarding the binding sites available on the FibBLG surface and their correlation with the percentage of Fe.

Given that BLG fibrils are generated through the random self-assembly of peptides and lack a definitive molecular weight, a direct calculation of the binding sites on the protein surface may not be possible. Nevertheless, we have endeavored to perform the calculation under ideal conditions, aiming to substantiate that BLG fibrils do, in fact, provide a greater number of binding sites than necessary. We hope this calculation helps address the concern of the reviewer. In this calculation, we utilized the same peptide fragment of BLG: LACQCL, Mw = 649.82 g mol^{-1}, employed in Molecular Dynamics (MD) simulation and Density Functional Theory (DFT) calculations.

Assuming that the BLG fibrils in Fe_{SA}@FibBLG are 100 mg, the amounts of peptides can be calculated as 100 mg/649.82 mg mmol-1 = 0.1539 mmol. If all the peptides are assembled into one fibril, the total amount of potential binding sites is of the order of 0.1539 mmol times the number of N sites contained in LACQCL, assumed to be 1 for simplicity (only one accessible N per repeat unit).

The Fe loading on BLG fibrils was determined to be 1.21% (w/w), equivalent to 100 mg * 1.21%/56 mg $mmol^{-1}$ = 0.02161 mmol, which is about 7 times lower than the estimated potentially accessible N sites.

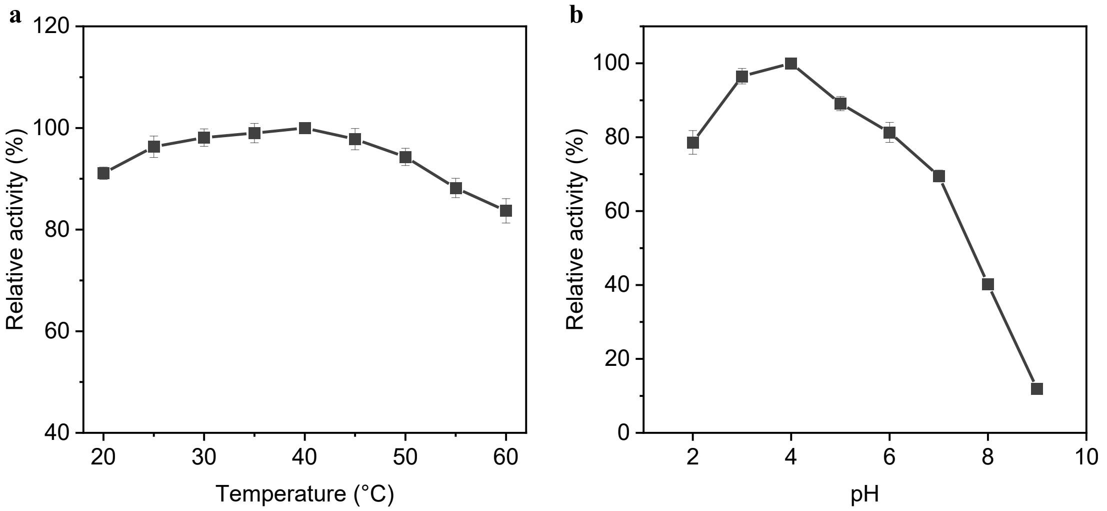

Supplementary Fig. 11. The effects of (a) temperature and (b) pH on the catalytic activity of Fe_{SA}@FibBLG. (n = 3 independent measurements, bars represent means ±s.d.)

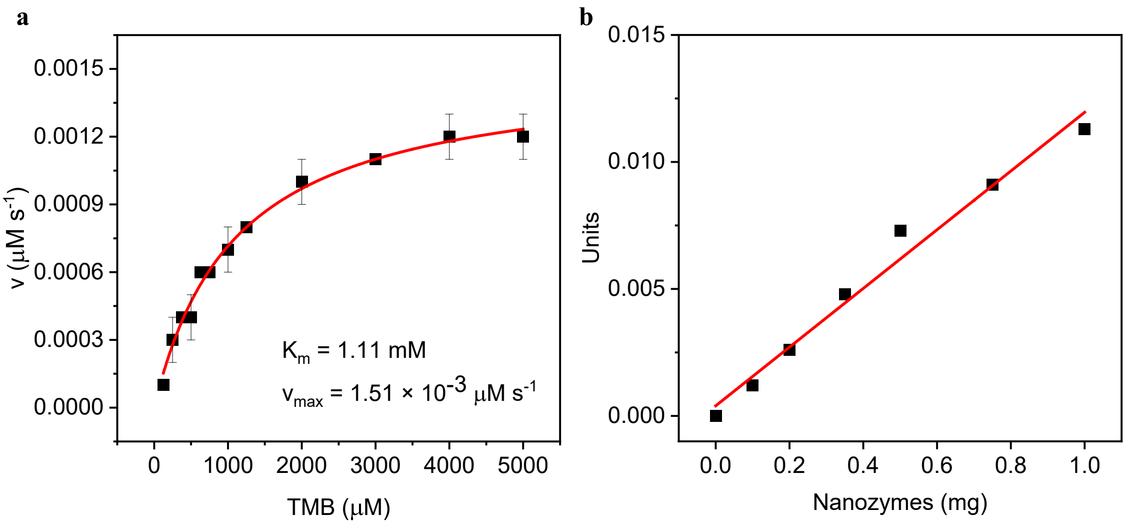

Supplementary Fig. 12. Characterization of the peroxidase-like activity and kinetics of BLG fibrils. a, The specific activities (U mg^{-1}) of FibBLG support. b, Michaelis-Menten curves fitted by ordinary Least square. The concentration of H_2O_2 used was 0.5 M, and the TMB concentration varied from 0 to nearly 5 mM. (n =3 independent measurements, bars represent means ± s.d.)

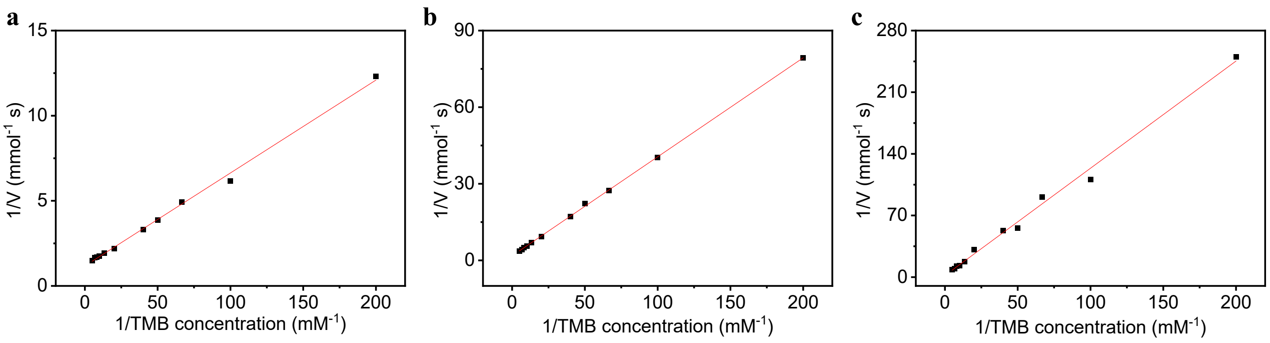

Supplementary Fig. 13. Kinetics parameters of Fe_{SA}@FibBLG, Fe_{SA}@BLG, FeNP@FibBLG for TMB substrate fitted by Lineweaver–Burk model.

Supplementary Table 3. Comparison of the kinetics based on Fe active sites anchored on Fe_{SA}@FibBLG, Fe_{SA}@BLG, FeNP@FibBLG for TMB substrate.

Nanozyme	[*E*] (M)	K_m (mM)	V_{max} (μM s^{-1})	K_{cat} (min^{-1})	K_{cat}/k_m (M^{-1} min^{-1})
Fe_{SA}@FibBLG	2.16×10^{-6}	4.00×10^{-2}	0.788	21.9	5.47×10^{8}
Fe_{SA}@BLG	1.95×10^{-6}	0.217	0.566	17.4	8.04×10^{7}
FeNP@FibBLG	2.64×10^{-6}	0.400	0.354	8.04	2.01×10^{7}

[E] is the molar concentration of the Fe in nanozymes, which was chosen to obtain the well-fitted Michaelis–Menten plots while varying the substrate concentrations. K_m is the Michaelis constant, V_{max} is the maximal reaction velocity and k_{cat} is the catalytic constant, where $k_{cat} = V_{max}/[E]$ and the k_{cat}/K_m value indicates the catalytic efficiency of these nanozymes.

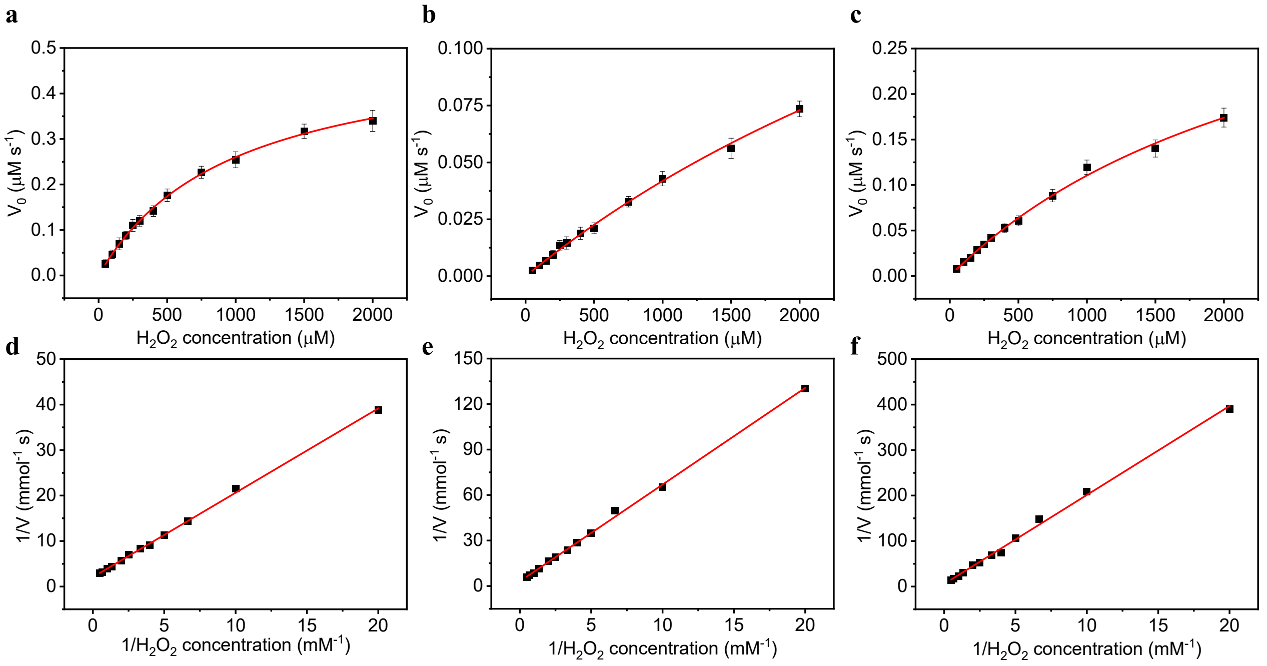

Supplementary Fig. 14. Kinetics parameters of Fe_{SA}@FibBLG, Fe_{SA}@BLG, FeNP@FibBLG for H_2O_2 substrate.

Supplementary Table 4. Comparison of the kinetics based on Fe active sites anchored on Fe_{SA}@FibBLG, Fe_{SA}@BLG, FeNP@FibBLG for H_2O_2 substrate.

Nanozyme	[*E*] (M)	K_m (mM)	V_{max} (μM s^{-1})	K_{cat} (min^{-1})	K_{cat}/k_m (M^{-1} min^{-1})
Fe_{SA}@FibBLG	2.16×10^{-6}	0.980	0.515	14.3	14.6×10^6
Fe_{SA}@BLG	1.95×10^{-6}	2.72	0.410	12.6	4.65×10^6
FeNP@FibBLG	2.64×10^{-6}	5.76	0.283	6.42	1.12×10^6

[E] is the molar concentration of the Fe in nanozymes, which was chosen to obtain the well-fitted Michaelis–Menten plots while varying the substrate concentrations. K_m is the Michaelis constant, V_{max} is the maximal reaction velocity and k_{cat} is the catalytic constant, where $k_{cat} = V_{max}/[E]$ and the k_{cat}/K_m value indicates the catalytic efficiency of these nanozymes.

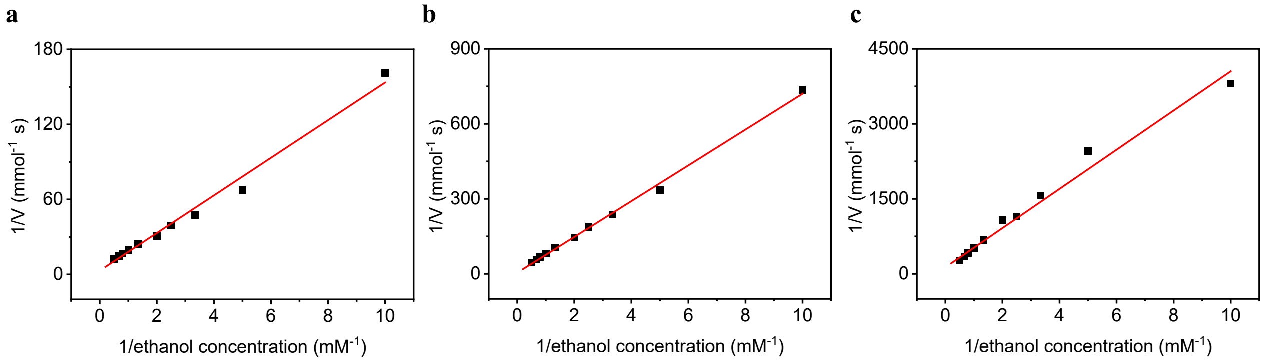

Supplementary Fig. 15. Kinetics parameters of Fe_{SA}@FibBLG, Fe_{SA}@BLG, FeNP@FibBLG for ethanol substrate fitted by Lineweaver–Burk model.

Supplementary Table 5. Comparison of the kinetics based on Fe active sites anchored on Fe_{SA}@FibBLG, Fe_{SA}@BLG, FeNP@FibBLG for ethanol substrate.

Nanozyme	[*E*] (M)	K_m (mM)	V_{max} (μM s^{-1})	K_{cat} (min^{-1})	K_{cat}/k_m (M^{-1} min^{-1})
Fe_{SA}@FibBLG	1.08×10^{-5}	2.39	0.177	0.983	4.11×10^5
Fe_{SA}@BLG	9.73×10^{-6}	7.55	0.106	0.654	8.66×10^4
FeNP@FibBLG	1.32×10^{-6}	18.7	3.81×10^{-2}	0.173	9.25×10^3

[E] is the molar concentration of the Fe in nanozymes, which was chosen to obtain the well-fitted Michaelis–Menten plots while varying the substrate concentrations. K_m is the Michaelis constant, V_{max} is the maximal reaction velocity and k_{cat} is the catalytic constant, where $k_{cat} = V_{max}/[E]$ and the k_{cat}/K_m value indicates the catalytic efficiency of these nanozymes.

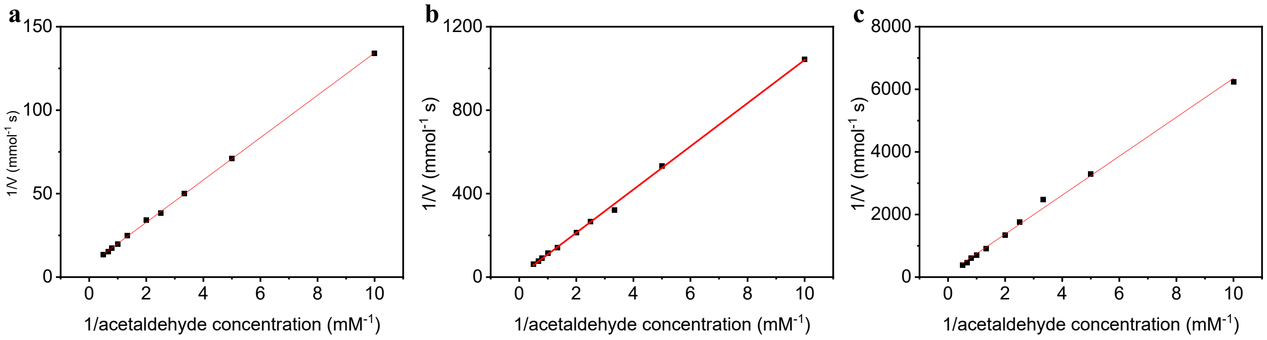

Supplementary Fig. 16. Kinetics parameters of Fe_{SA}@FibBLG, Fe_{SA}@BLG, FeNP@FibBLG for acetaldehyde substrate fitted by Lineweaver–Burk model.

Supplementary Table 6. Comparison of the kinetics based on Fe active sites anchored on Fe_{SA}@FibBLG, Fe_{SA}@BLG, FeNP@FibBLG for acetaldehyde substrate.

Nanozyme	[*E*] (M)	K_m (mM)	V_{max} (μM s^{-1})	K_{cat} (min^{-1})	K_{cat}/k_m (M^{-1} min^{-1})
Fe_{SA}@FibBLG	1.08×10^{-5}	2.27	0.159	0.883	3.89×10^{5}
Fe_{SA}@BLG	9.73×10^{-6}	7.88	8.04×10^{-2}	0.496	6.29×10^{4}
FeNP@FibBLG	1.32×10^{-6}	12.6	1.91×10^{-2}	8.67×10^{-2}	6.88×10^{3}

[E] is the molar concentration of the Fe in nanozymes, which was chosen to obtain the well-fitted Michaelis–Menten plots while varying the substrate concentrations. K_m is the Michaelis constant, V_{max} is the maximal reaction velocity and k_{cat} is the catalytic constant, where $k_{cat} = V_{max}/[E]$ and the k_{cat}/K_m value indicates the catalytic efficiency of these nanozymes.

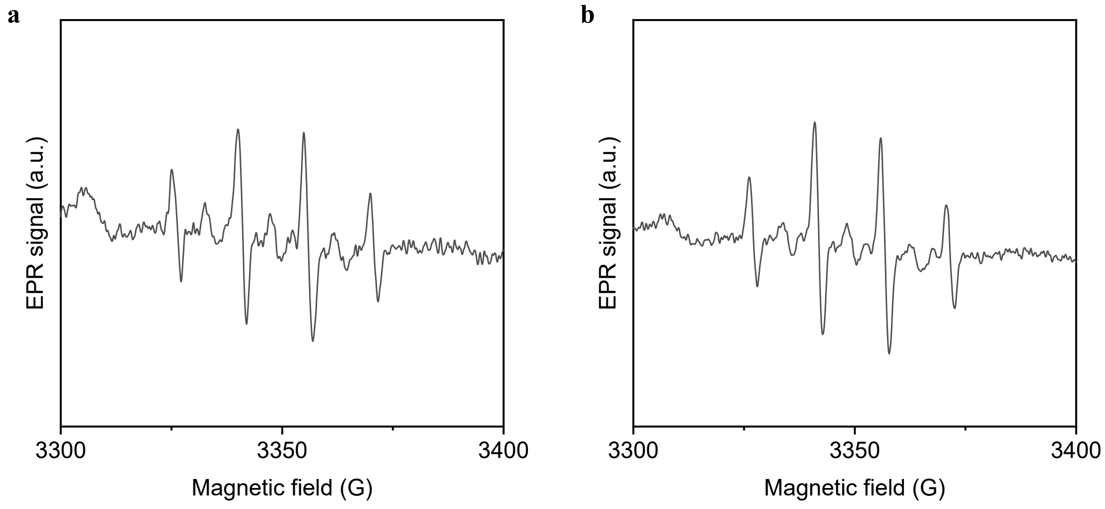

Supplementary Fig. 17. EPR Spectra of DMPO/H_2O_2/ethanol (a) and DMPO/H_2O_2/acetaldehyde (b) reaction systems upon the addition of Fe_{SA}@FibBLG.

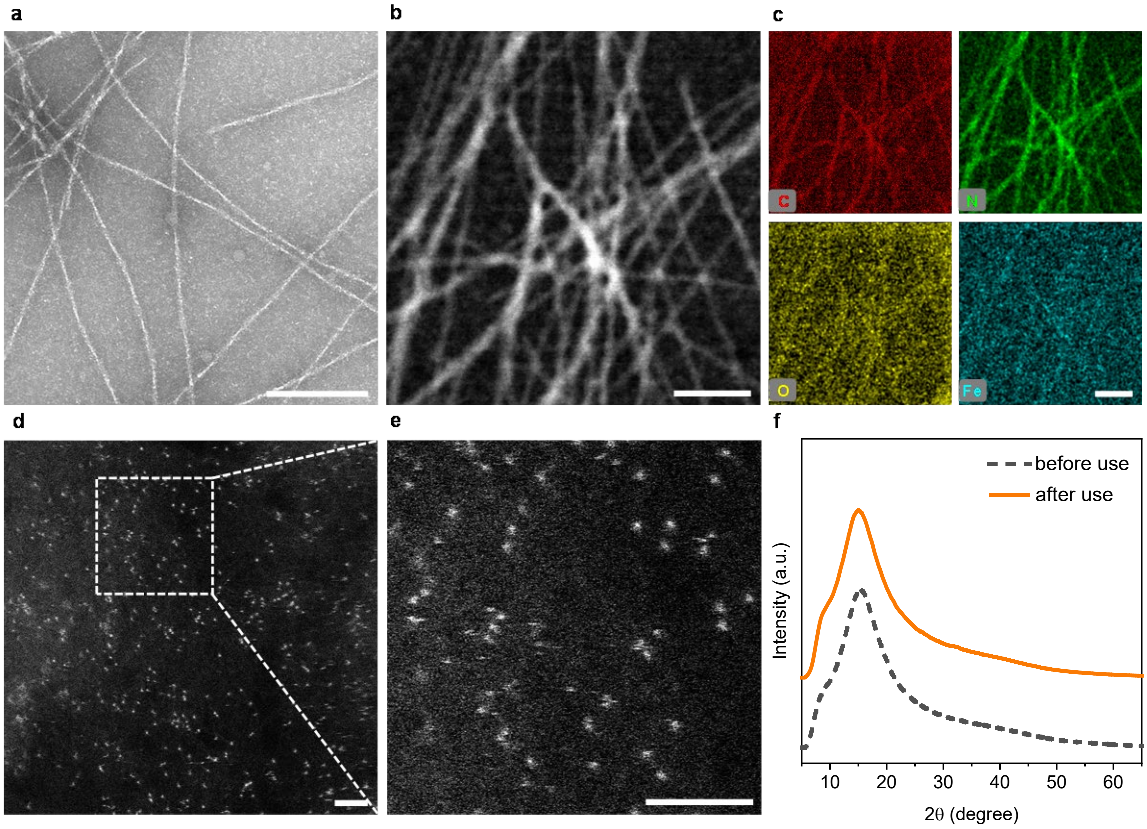

Supplementary Fig. 18. a-c, TEM image (a), HR-TEM image (b), and the corresponding EDS element maps (c) of Fe_{SA}@FibBLG after the catalytic test. Scale bars, 200 nm. d,e, The aberration-corrected HAADF-STEM image (d) and enlarged aberration-corrected HAADF-STEM image (e) of Fe@FibBLG after catalytic reaction. Scale bars, 2 nm. In a-e, images illustrate results consistent across six technical replicates (n=6), demonstrating reproducible outcomes. f, XRD patterns of Fe_{SA}@FibBLG before and after the catalytic test.

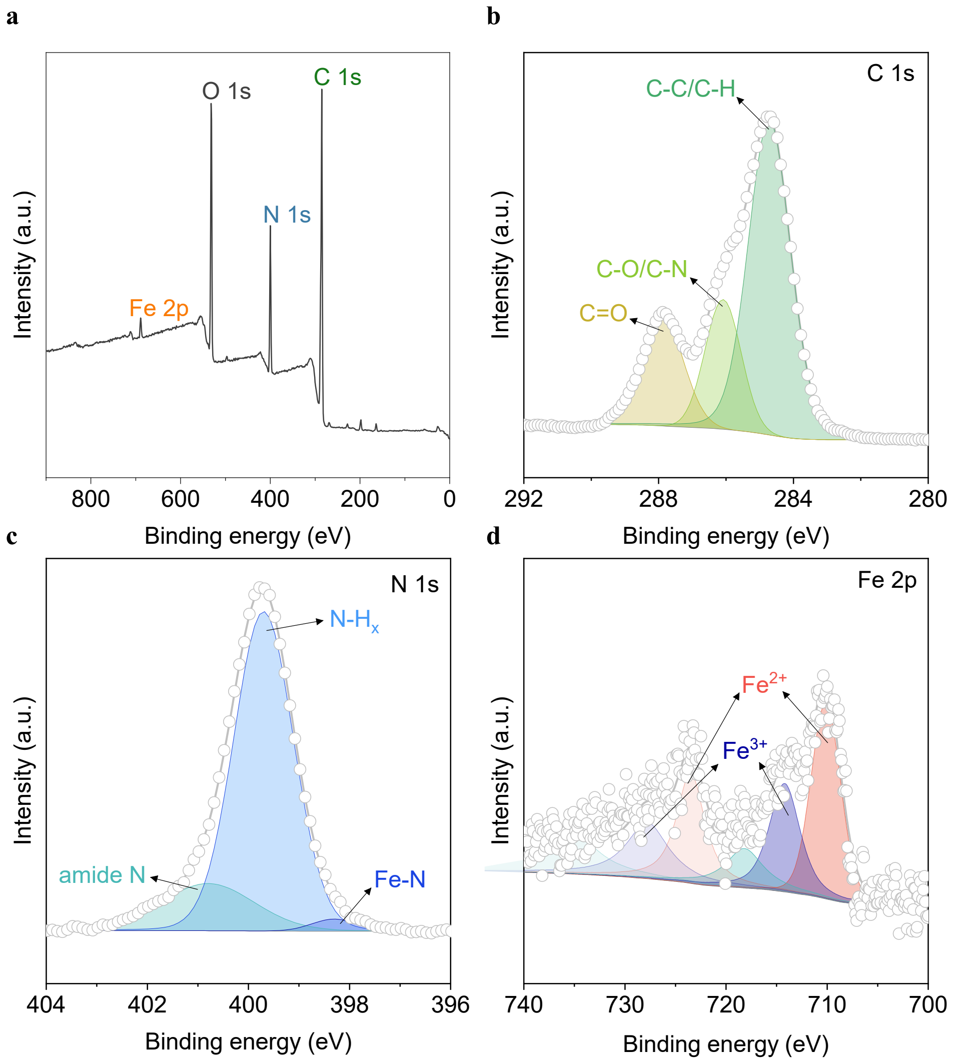

Supplementary Fig. 19. XPS survey (a) and the deconvoluted C 1s (b), N 1s (c), and Fe 2p (d) spectra of Fe_{SA}@FibBLG after catalytic reaction.

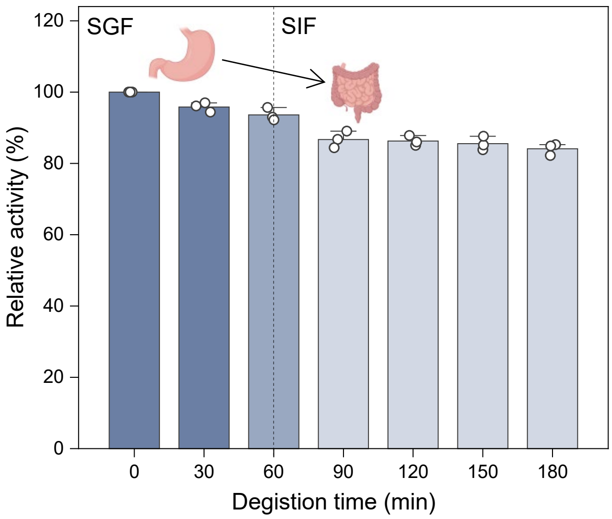

Supplementary Fig. 20. The effect of *in vitro* digestion process on the catalytic activity of Fe_{SA}@FibBLG. SGF: simulated gastric fluid. SIF: Simulated intestinal fluid. (n =3 independent measurements, bars represent means ± s.d.)

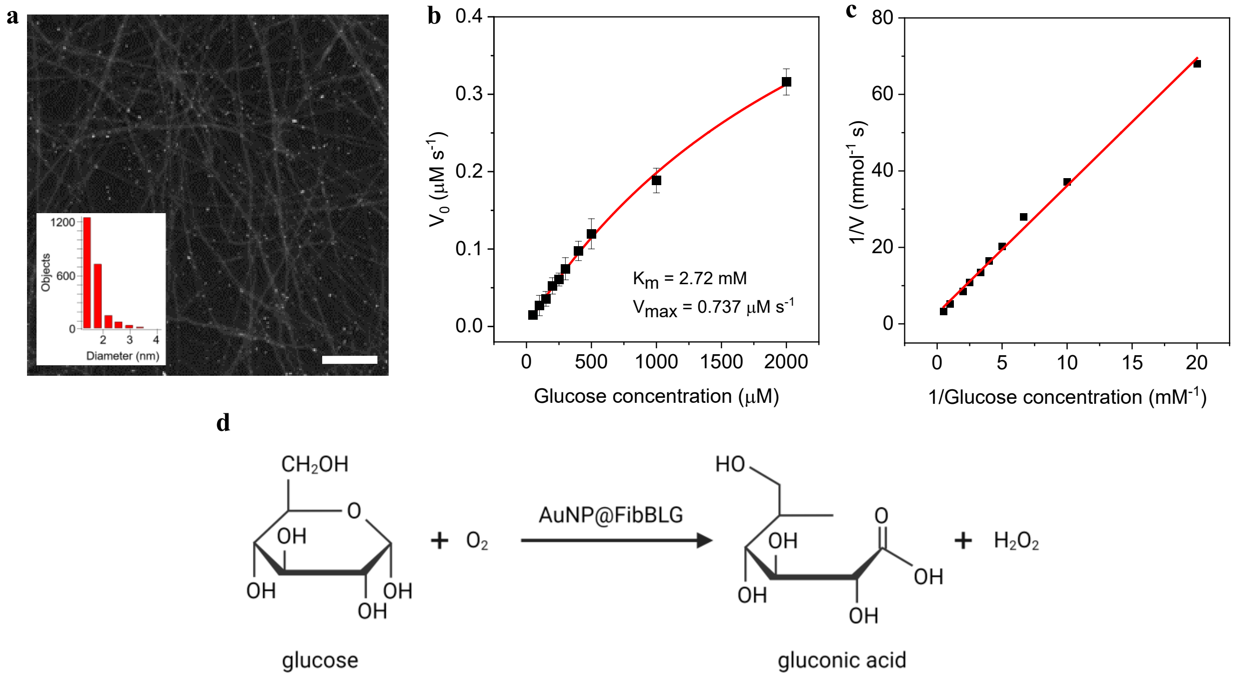

Supplementary Fig. 21. a, STEM images of AuNP@FibBLG and particle size distribution of AuNPs (inserted a). Scale bars, 200 nm. Images illustrate results consistent across six technical replicates (n=6), demonstrating reproducible outcomes. b,c, Steady-state kinetic analysis of AuNP@FibBLG for glucose by using (b) Michaelis–Menten model and (c) Lineweaver–Burk plots. These analyses aim to quantify the catalytic efficiency of H_2O_2 production within the reaction system. d, Illustration of the AuNP@FibBLG-based catalytic reaction for H_2O_2 production.

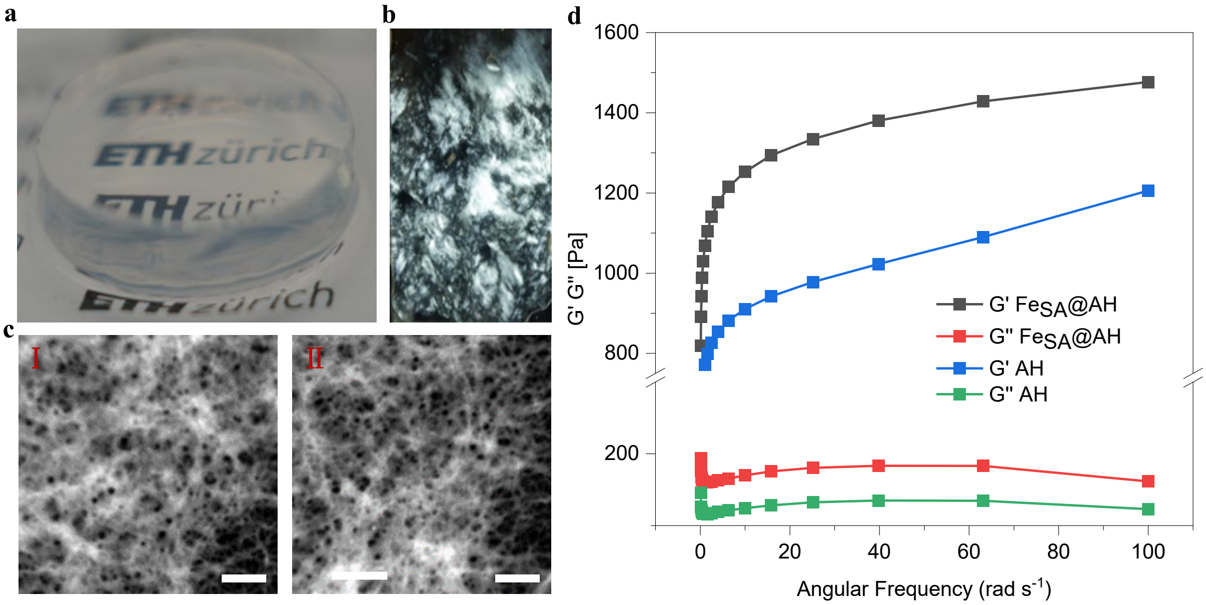

Supplementary Fig. 22. Visual observation of (a) Na^+-induced AuNPFe$_{SA}$@FibBLG amyloid hydrogel (Fe$_{SA}$@AH) and its microstructure under polarized light (b). c, STEM images of AH (I) and Fe$_{SA}$@AH (II) Scale bars, 20 nm. Images illustrate results consistent across six technical replicates (n=6), demonstrating reproducible outcomes. d, Rheological properties of AH and Fe$_{SA}$@AH.

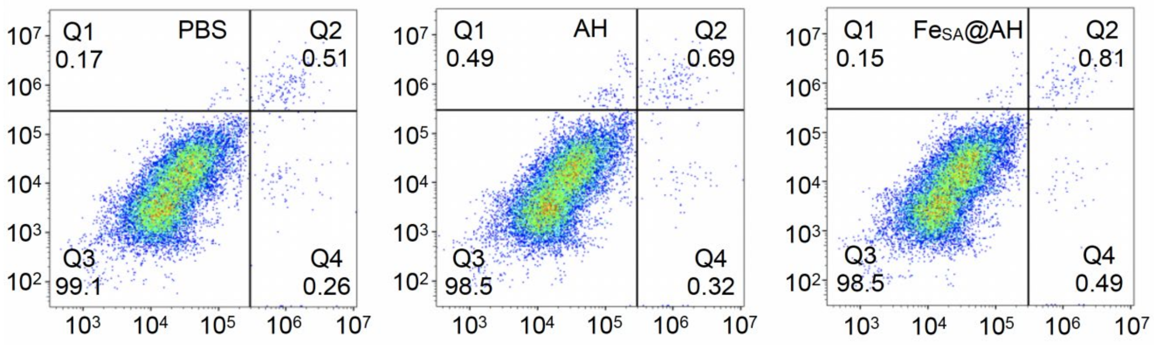

Supplementary Figure 23. Cytotoxicity evaluation of nanozymes. Representative images of FITC/PI double-staining analysis in Caco-2 cells treated with various samples by flow cytometry. Bottom right quadrant, FITC 9(+)/PI(−), early apoptotic cells; top right quadrant, FITC(+)/PI(+), necrotic cells; bottom lift quadrant, FITC(−)/PI(−), viable cells.

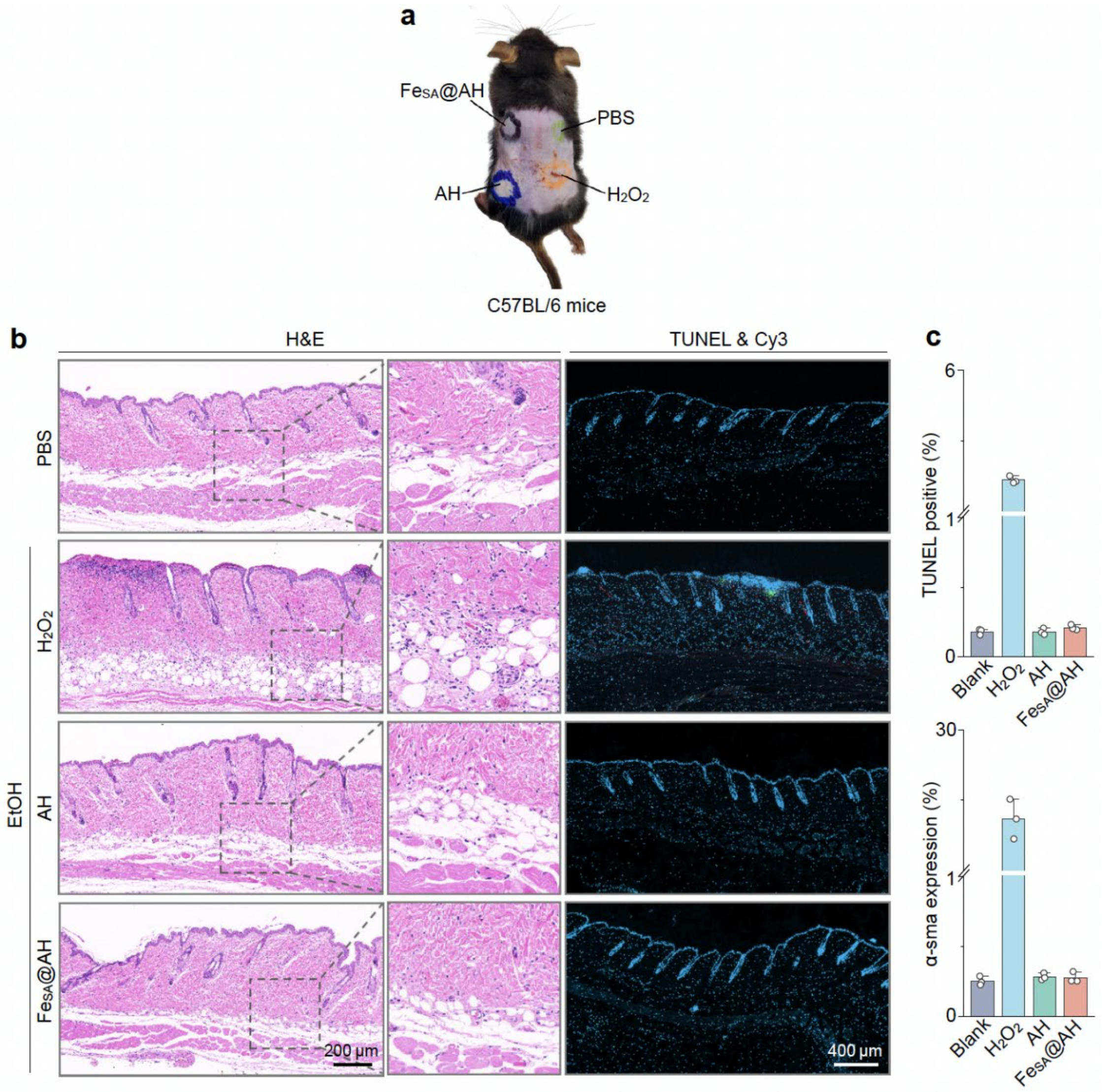

Supplementary Figure 24. Allergenicity test of nanozymes on mice skin. a, Photograph of a mouse cutaneously injected with PBS, Fe$_{SA}$@AH, AH, and H_2O_2 at different sites. b, Micrographs of mouse skin tissue at the injection sites. Left: H&E-stained images. Right: Immunohistology stain with TUNEL assay (green), Cy3-conjugated monoclonal α-smooth muscle actin antibody (red), and DAPI (blue). c, the relative intensity of TUNEL fluorescence and α-sma expression. In figure b, images displayed are representative of three technical replicates (n=3), each producing consistent results. Data in figure c are shown in the form of mean ± SEM, n = 3 technical replicates. One-way ANOVA followed by Tukey's multiple-range test was used.

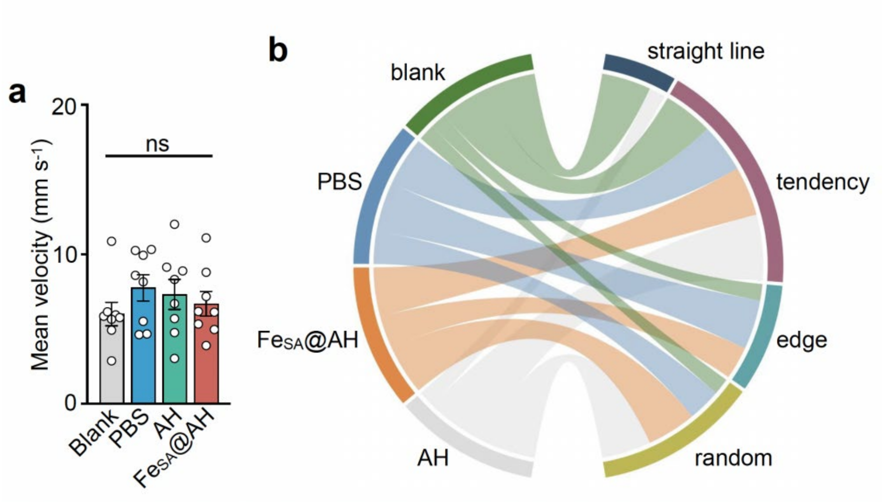

Supplementary Figure 25. Effect of different treatments (PBS, AH, and Fe_{SA}@AH) on the mean velocity (a) and search strategies (b) of mice to locate the hidden platform in Morris water maze tests. Data are shown in the form of mean ± SEM, n = 8 independent biological replications. One-way ANOVA followed by Tukey's multiple-range test was used. ns, no significance.

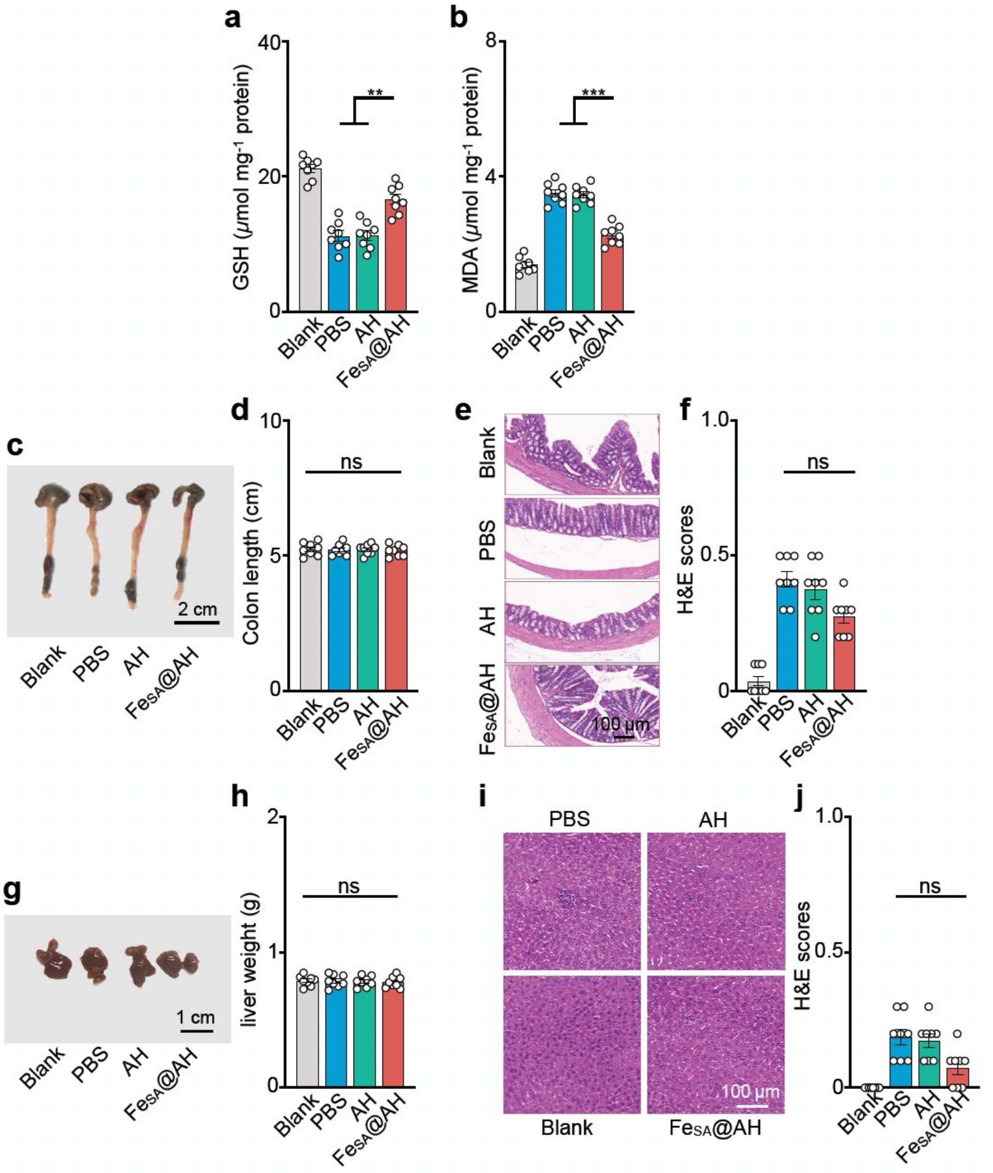

Supplementary Figure 26. Histopathological, physiological, and biochemical alterations in mice exposed to acute alcohol intoxication treated with PBS, AH, and Fe_{SA}@AH. a,b, Biochemical measurements of GSH (a) and MDA (b) in liver tissue. c,d, Representative colon images (c) of mice in each group and corresponding quantification for colon length (d). e,f, Representative H&E-stained images (e) and scores (f) of colonic tissues. g,h, Representative liver images (g) of mice in each group and corresponding quantification for the liver weight (h). i,j, H&E-stained images (i) and scores (j) of liver sections. In figures e and i, images displayed are representative of three biological replicates (n=3), each producing consistent results. In figures a, b, d, f, h, and j, data are shown in the form of mean ± SEM, n = 8 independent biological replications. One-way ANOVA followed by Tukey's multiple-range test. * $p<0.05$, ** $p<0.01$, *** $p<0.001$, **** $p<0.0001$. ns, no significance.

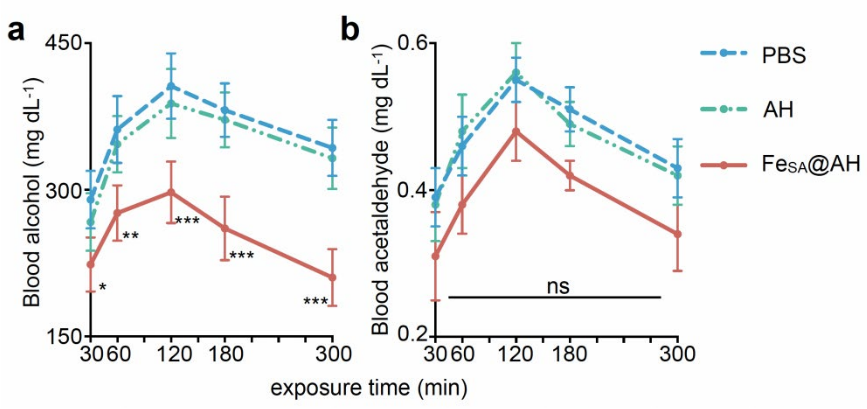

Supplementary Figure 27. Blood alcohol concentrations (a) and blood acetaldehyde concentrations (b) in chronic alcohol-intoxicated mice treated with PBS, AH, and Fe_{SA}@AH. Data are shown in the form of mean ± SEM, n = 8 independent biological replications. One-way ANOVA followed by Tukey's multiple-range test was used. * p<0.05, ** p<0.01, *** p<0.001, **** p<0.0001. ns, no significance.

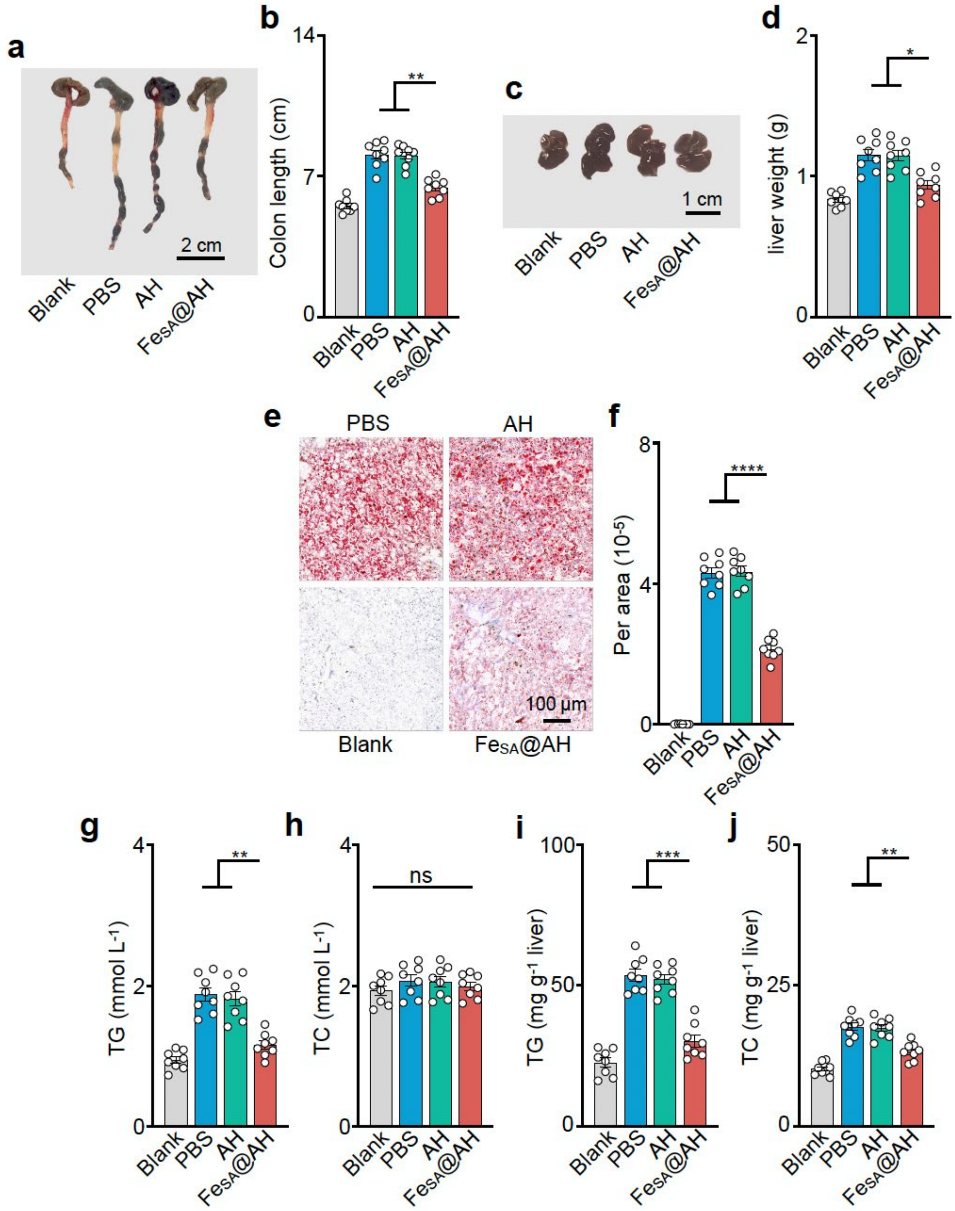

Supplementary Figure 28. Histopathological, physiological, and biochemical alterations in mice exposed to chronic alcohol intoxication treated with PBS, AH, and Fe$_{SA}$@AH. a,b, Representative colon images (a) of mice in each group and corresponding quantification for colon length (b). c,d, Representative H&E-stained images (c) and scores (d) of colonic tissues. e,f, Oil red O staining (e) and scores (f) of liver sections. g,h, serum TG (c) and serum total cholesterol (d) in blood. i,j, liver triglyceride (TG) content (b) and liver total cholesterol. In figure e, images displayed are representative of three biological replicates (n=3), each producing consistent results. Data are shown in the form of mean ± SEM, n = 8 independent biological replications. One-way ANOVA followed by Tukey's multiple-range test was used. * $p<0.05$, ** $p<0.01$, *** $p<0.001$, **** $p<0.0001$. ns, no significance.

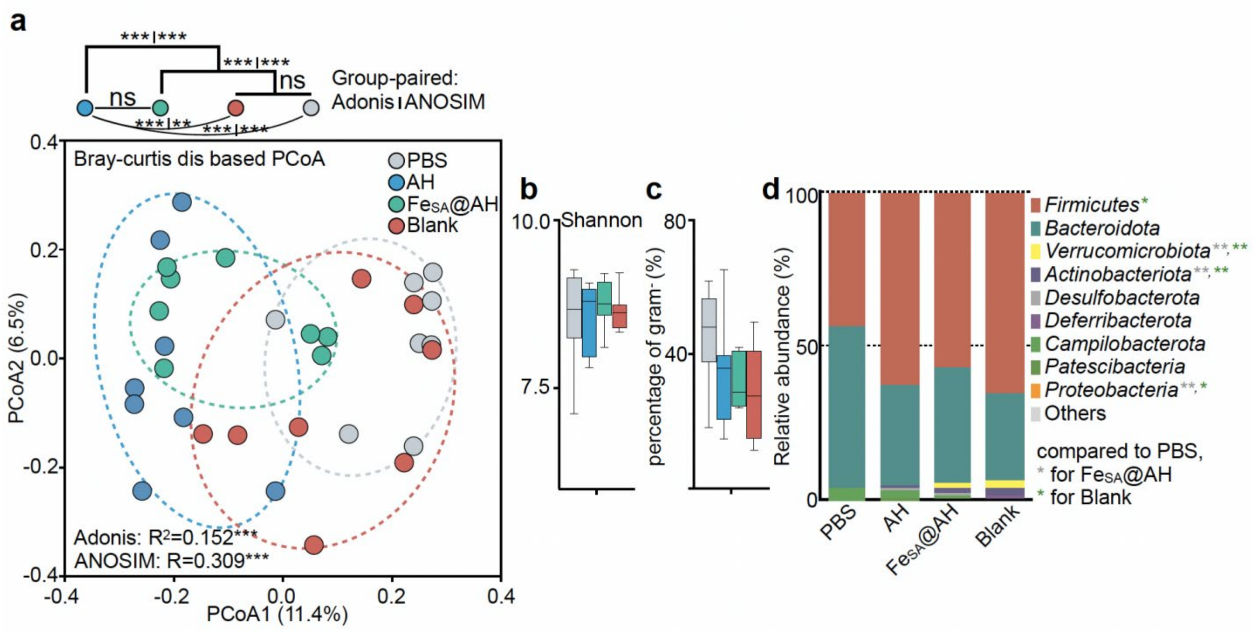

Supplementary Figure 29. a, Principal coordinate analysis (PCoA) result with Bray−Curtis distances and compositional shifts, the differences in beta-diversity, among groups are tested by both PERMANOVA (Adonis) and ANOSIM. *** $p < 0.001$. b,c, Gut microbiota α diversity, the Shannon index (b), and the gross percentages of Gram-negative ($Gram^-$) bacteria (c) among 4 groups, median with minima to maxima and bounds of box and whiskers indicate the 25% and 75% percentiles. d, Mean relative abundance of the top 9 bacterial phyla, selectively from the PBS, in each group. n=8 independent biological replications. Paired-wise Wilcoxon test with Bonferroni-Holm correction: * $p < 0.05$, ** $p < 0.01$. ns, no significance.

Supplementary Table 7. Standard of histological scoring of mice skin upon injection treatments of PBS, AH, Fe_{SA}@AH, and H_2O_2.

Histological features	Score	Description
Epithelium loss	0	None
	1	0.0-5.0%
	2	5.0-10.0%
	3	Over 10.0%
Crypt damage	0	None
	1	0.0-10.0%
	2	10.0-20.0%
	3	Over 20.0%
Depletion of goblet cells	0	None
	1	Mild
	2	Moderate
	3	Severe

Supplementary Table 8. Standard of histological scoring of mice colon upon alcohol administration and gavage treatments of PBS, AH and Fe_{SA}@AH.

Histological features	Score	Description
Inflammation severity	0	None
	1	Minimal
	2	Mild
	3	Moderate
	4	Severe
Crypt damage	0	None
	1	Basal 1/3 damaged
	2	Basal 2/3 damaged
	3	Crypts lost; surface epithelium present
	4	Crypts lost and surface epithelium lost
Epithelial injury	0	None
	1	Minimal
	2	Mild
	3	Moderate
	4	Severe
Muscular injury	0	None
	1	Minimal
	2	Mild
	3	Moderate
	4	Severe
Fibrous connective tissue proliferation	0	None
	1	Minimal
	2	Mild
	3	Moderate
	4	Severe

Supplementary Table 9. Standard of histological scoring of mice liver upon alcohol administration and gavage treatments of PBS, AH and Fe_{SA}@AH.

Histological features	Score	Description
Steatosis	0	Under 5%
	1	5-33%
	2	34-66%
	3	Over 66%
Lobular inflammation	0	No foci
	1	1-2 foci per ×20 field
	2	2-4 foci per ×20 field
	3	Over 4 foci per ×20 field
Hepatocellular ballooning	0	None
	1	Moderate
	2	Evident

Supplementary Table 10. Summarized statistical results of microbial function shifts in AH, Fe_{SA}@AH, and blank compared to the group PBS.

KEGG-id	annotations	grouped mean abundance				KEGGid	adjusted p values compared to group PBS			
		PBS	blank	AH	Fe_{SA}@AH		group	p	p.adj	p.adj.signif
ko00010	Glycolysis / Gluconeogenesis	24512.76013	18662.18841	14618.42679	15041.8645	ko00020	Fe_{SA}@AH	0.002837545	0.008512635	**
ko00020	Citrate cycle (TCA cycle)	18978.52935	12744.48868	7014.955638	8170.53195	ko00020	blank	0.000289619	0.001737717	**
ko00030	Pentose phosphate pathway	35949.74801	28823.41325	20997.51223	21508.89851	ko00030	Fe_{SA}@AH	0.010515246	0.031545738	*
ko00040	Pentose and glucuronate interconversions	14307.11558	12042.36118	7202.835438	7941.70635	ko00030	blank	0.007109759	0.031545738	*
ko00051	Fructose and mannose metabolism	24418.84208	17398.51421	14114.41254	14274.79245	ko00040	Fe_{SA}@AH	0.010515246	0.021030492	*
ko00052	Galactose metabolism	23284.06251	18481.06565	15542.92081	15929.81036	ko00040	blank	0.00126122	0.007567319	**
ko00053	Ascorbate and aldarate metabolism	4093.475913	3576.35015	1781.692713	1933.187163	ko00053	Fe_{SA}@AH	0.020419026	0.046993751	*
ko00061	Fatty acid biosynthesis	24530.41154	20837.61711	9806.914613	11447.59154	ko00053	blank	0.007109759	0.042658554	*
ko00071	Fatty acid metabolism	5939.20765	5209.337838	3423.345163	3682.4931	ko00061	Fe_{SA}@AH	0.00656165	0.01968495	*
ko00072	Synthesis and degradation of ketone bodies	8772.691563	7563.0775	6046.19625	5708.679063	ko00061	blank	0.001148742	0.006892449	**
ko00100	Steroid biosynthesis	0.0522	0.020775	51.7446875	56.869675	ko00071	Fe_{SA}@AH	0.013195152	0.039585457	*
ko00120	Primary bile acid biosynthesis	4136.116663	2365.314163	3294.50695	3453.81765	ko00071	blank	0.00656165	0.039369901	*
ko00121	Secondary bile acid biosynthesis	37225.05	21287.8275	29650.5625	31084.35875	ko00130	Fe_{SA}@AH	0.008330837	0.024992511	*
ko00130	Ubiquinone and other terpenoid-quinone biosynthesis	7817.522125	4723.770813	3094.871375	3671.203688	ko00130	blank	0.001045598	0.00627359	**
ko00140	Steroid hormone biosynthesis	497.5094	441.0514375	280.5273625	407.427425	ko00190	Fe_{SA}@AH	0.003674177	0.011022531	*
ko00190	Oxidative phosphorylation	8961.874663	6354.80695	3673.474963	4185.3679	ko00190	blank	0.000435121	0.002610725	**
ko00230	Purine metabolism	17434.75583	12872.93121	9378.1979	9864.029325	ko00230	Fe_{SA}@AH	0.004729397	0.014188192	*
ko00240	Pyrimidine metabolism	23943.03376	16670.71946	12516.83916	13295.21965	ko00230	blank	0.002380669	0.014188192	*
ko00250	Alanine, aspartate and glutamate metabolism	35035.11275	26734.55273	19125.10708	20888.13861	ko00240	Fe_{SA}@AH	0.00656165	0.01968495	*
ko00260	Glycine, serine and threonine metabolism	19243.61876	16610.40724	9476.35085	10377.61375	ko00240	blank	0.004350533	0.01968495	*
ko00270	Cysteine and methionine metabolism	23261.76801	18913.73311	12404.20983	13243.9514	ko00250	Fe_{SA}@AH	0.003674177	0.011022531	*
ko00280	Valine, leucine and isoleucine degradation	5566.636825	5703.234825	3479.113263	3733.785775	ko00250	blank	0.000712921	0.004277525	**
ko00281	Geraniol degradation	1393.3086	1089.343438	647.109225	873.709525	ko00260	Fe_{SA}@AH	0.013195152	0.026390305	*
ko00290	Valine, leucine and isoleucine biosynthesis	39184.81226	34881.67284	21716.71216	23302.06331	ko00260	blank	0.001045598	0.00627359	**
ko00300	Lysine biosynthesis	29859.15381	25282.82213	17025.50088	17697.5275	ko00270	Fe_{SA}@AH	0.002837545	0.008512635	**

ko00310	Lysine degradation	2633.236713	2934.434538	1568.25255	1660.020725	ko00270	blank	0.000393415	0.002360492	**
ko00311	Penicillin and cephalosporin biosynthesis	440.5107	116.675	95.95715	89.521425	ko00280	Fe_{SA}@AH	0.002837545	0.008512635	**
ko00312	beta-Lactam resistance	5034.59625	5615.058125	2053.504375	1883.763125	ko00280	blank	0.000785338	0.004712029	**
ko00330	Arginine and proline metabolism	13382.60248	11399.58045	6790.596688	7527.432963	ko00290	Fe_{SA}@AH	0.007698627	0.015397255	*
ko00340	Histidine metabolism	22558.73995	20530.21776	12774.18179	13672.53298	ko00290	blank	0.000646749	0.003880495	**
ko00350	Tyrosine metabolism	4907.019025	3658.935563	2663.814463	2780.975713	ko00300	Fe_{SA}@AH	0.002178445	0.006535336	**
ko00360	Phenylalanine metabolism	4504.561688	3832.159125	2515.906638	2575.879238	ko00300	blank	0.000480925	0.00288555	**
ko00361	Chlorocyclohexane and chlorobenzene degradation	651.2045	1401.03125	962.87875	874.377	ko00310	Fe_{SA}@AH	0.00656165	0.01968495	*
ko00362	Benzoate degradation	3028.21475	2538.175788	2004.7627	1998.654575	ko00310	blank	0.001383791	0.008302747	**
ko00380	Tryptophan metabolism	2931.142588	3455.050188	0	645.1847875	ko00311	Fe_{SA}@AH	0.004729397	0.028376384	*
ko00400	Phenylalanine, tyrosine and tryptophan biosynthesis	20266.7605	18198.56436	9981.34995	11275.41964	ko00330	Fe_{SA}@AH	0.020419026	0.040838052	*
ko00430	Taurine and hypotaurine metabolism	6584.750613	5728.420775	6685.009213	5843.489688	ko00330	blank	0.001148742	0.006892449	**
ko00440	Phosphonate and phosphinate metabolism	2394.966763	2937.924113	1897.087763	2212.319388	ko00340	Fe_{SA}@AH	0.017698893	0.030628539	*
ko00450	Selenocompound metabolism	18290.16559	14498.55866	9782.827175	10378.98	ko00340	blank	0.002599946	0.009284445	**
ko00471	D-Glutamine and D-glutamate metabolism	43581.34125	33081.56416	23796.1275	25201.78418	ko00350	Fe_{SA}@AH	0.003999392	0.011998177	*
ko00472	D-Arginine and D-ornithine metabolism	1668.17	4501.6	447.63125	450.17625	ko00350	blank	0.003373187	0.011998177	*
ko00473	D-Alanine metabolism	33509.26041	23536.86375	16313.78	17824.97251	ko00360	Fe_{SA}@AH	0.008330837	0.024992511	*
ko00480	Glutathione metabolism	9411.018538	5265.555175	4234.826063	4592.911125	ko00360	blank	0.008330837	0.024992511	*
ko00500	Starch and sucrose metabolism	21871.48009	15781.86933	11840.71916	12482.57026	ko00361	AH	0.007698627	0.046191764	*
ko00510	N-Glycan biosynthesis	1485.7385	916.1182375	619.31375	758.600125	ko00380	Fe_{SA}@AH	0.002003906	0.004007812	**
ko00511	Other glycan degradation	33774.37094	17961.61576	15302.84186	18582.06828	ko00380	blank	0.000100388	0.000602328	***
ko00520	Amino sugar and nucleotide sugar metabolism	23746.93194	17683.1374	14919.89044	15166.6794	ko00400	blank	0.005137883	0.030827299	*
ko00521	Streptomycin biosynthesis	34049.54489	24071.26389	19263.8393	20631.10665	ko00450	Fe_{SA}@AH	0.003674177	0.011022531	*
ko00531	Glycosaminoglycan degradation	12845.88966	5911.893025	5909.540188	8025.479838	ko00450	blank	0.000864534	0.005187207	**
ko00540	Lipopolysaccharide biosynthesis	15948.20109	7389.082413	4215.678588	5687.930838	ko00471	Fe_{SA}@AH	0.004729397	0.014188192	*
ko00550	Peptidoglycan biosynthesis	37396.87395	28478.88913	19979.68375	20953.2354	ko00471	blank	0.004350533	0.014188192	*
ko00561	Glycerolipid metabolism	9109.768488	8414.3827	5523.208188	5770.987563	ko00480	Fe_{SA}@AH	0.013195152	0.039585457	*
ko00562	Inositol phosphate metabolism	3976.125588	3549.323325	1749.061363	1954.50195	ko00480	blank	0.010515246	0.039585457	*
ko00564	Glycerophospholipid metabolism	11059.98745	8487.808213	6218.055338	6586.281688	ko00500	Fe_{SA}@AH	0.003094815	0.009284445	**

ko00600	Sphingolipid metabolism	9314.9995	3795.206063	2362.485813	4862.100438	ko00500	blank	0.000785338	0.004712029	**
ko00601	Glycosphingolipid biosynthesis - lacto and neolacto series	11.6827	5.0769125	0.0384625	11.97115	ko00511	blank	0.002380669	0.014284012	*
ko00620	Pyruvate metabolism	20976.71906	17890.57391	11652.20458	12449.55925	ko00511	AH	0.015301719	0.045905158	*
ko00627	Aminobenzoate degradation	2174.595838	1476.969175	1378.715013	1401.252	ko00521	Fe_{SA}@AH	0.003999392	0.011998177	*
ko00630	Glyoxylate and dicarboxylate metabolism	10795.4338	9996.56755	6317.863325	6701.822175	ko00521	blank	0.001148742	0.006892449	**
ko00633	Nitrotoluene degradation	3980.130675	3574.859988	1804.462625	2157.99715	ko00540	blank	0.003094815	0.01856889	*
ko00640	Propanoate metabolism	9761.367613	9344.636213	6573.846588	6828.987963	ko00550	Fe_{SA}@AH	0.003373187	0.01011956	*
ko00650	Butanoate metabolism	12364.29254	9969.0982	6607.107238	6849.844825	ko00550	blank	0.003373187	0.01011956	*
ko00660	C5-Branched dibasic acid metabolism	31337.06063	26421.82543	15766.3425	17143.46834	ko00561	Fe_{SA}@AH	0.009009096	0.027027288	*
ko00670	One carbon pool by folate	34991.13073	25093.98446	17845.14983	19379.77221	ko00561	blank	0.002380669	0.014284012	*
ko00680	Methane metabolism	9267.0551	7159.665075	4468.802575	4856.145788	ko00562	Fe_{SA}@AH	0.003094815	0.009284445	**
ko00710	Carbon fixation in photosynthetic organisms	35284.37834	26037.42083	18870.945	19721.69251	ko00562	blank	0.000480925	0.00288555	**
ko00720	Carbon fixation pathways in prokaryotes	20968.74645	14860.27329	9336.611613	10496.69339	ko00564	Fe_{SA}@AH	0.009009096	0.027027288	*
ko00730	Thiamine metabolism	26426.11295	22673.54114	14860.31079	16565.02035	ko00564	blank	0.00656165	0.027027288	*
ko00740	Riboflavin metabolism	13300.18905	9572.6924	4628.019263	5874.2851	ko00600	blank	0.001326906	0.007961435	**
ko00750	Vitamin B6 metabolism	20692.58938	14531.49374	8447.667825	9938.237025	ko00620	Fe_{SA}@AH	0.007109759	0.021329277	*
ko00760	Nicotinate and nicotinamide metabolism	21523.82911	15085.13001	10239.26598	11175.85329	ko00620	blank	0.001992081	0.011952486	*
ko00770	Pantothenate and CoA biosynthesis	32394.89125	26643.05791	16606.86633	18171.2195	ko00630	Fe_{SA}@AH	0.005137883	0.015413649	*
ko00780	Biotin metabolism	19840.64725	15524.8535	9215.93825	12352.9785	ko00630	blank	0.000864534	0.005187207	**
ko00785	Lipoic acid metabolism	18568.16625	13804.99375	5171.1625	6225.411875	ko00650	Fe_{SA}@AH	0.005137883	0.023095882	*
ko00790	Folate biosynthesis	22064.98313	14135.48916	8473.262713	10218.97449	ko00650	blank	0.007698627	0.023095882	*
ko00791	Atrazine degradation	555.5310625	276.8909	136.0100125	175.7941	ko00660	Fe_{SA}@AH	0.010515246	0.021030492	*
ko00830	Retinol metabolism	388.2372875	427.4325	0	0	ko00660	blank	0.000712921	0.004277525	**
ko00860	Porphyrin and chlorophyll metabolism	8893.225188	8341.577025	3342.179913	4092.291513	ko00670	Fe_{SA}@AH	0.003094815	0.009284445	**
ko00900	Terpenoid backbone biosynthesis	26944.78626	20798.31598	14582.31821	15372.3823	ko00670	blank	0.000586327	0.003517964	**
ko00906	Carotenoid biosynthesis	54.171625	773.289175	27.6739625	37.11515	ko00680	Fe_{SA}@AH	0.004350533	0.0130516	*
ko00908	Zeatin biosynthesis	15276.2825	9588.080625	7161.576563	7984.430938	ko00680	blank	0.000712921	0.004277525	**
ko00910	Nitrogen metabolism	11547.9878	9074.338875	5932.7945	6417.294425	ko00710	Fe_{SA}@AH	0.001992081	0.005976243	**
ko00920	Sulfur metabolism	12848.24243	11346.8021	6694.167888	7201.746725	ko00710	blank	0.000712921	0.004277525	**

ko00930	Caprolactam degradation	322.535725	25.6696375	141.63395	105.7499875	ko00720	Fe_{SA}@AH	0.001517266	0.004551799	**
ko00941	Flavonoid biosynthesis	8.35	30.975	1.175	3.15625	ko00720	blank	0.000435121	0.002610725	**
ko00960	Tropane, piperidine and pyridine alkaloid biosynthesis	7724.713225	4994.027075	3135.928225	1853.436875	ko00730	Fe_{SA}@AH	0.009736242	0.029208727	*
ko00970	Aminoacyl-tRNA biosynthesis	33058.12118	25136.1829	18493.43743	19406.18244	ko00730	blank	0.002178445	0.013070671	*
ko01040	Biosynthesis of unsaturated fatty acids	5393.439838	5095.886513	2953.440675	3116.1465	ko00740	Fe_{SA}@AH	0.007109759	0.021329277	*
ko01051	Biosynthesis of ansamycins	90849.21875	96125.20875	76218.6175	72127.24625	ko00740	blank	0.000154134	0.000924804	***
ko01053	Biosynthesis of siderophore group nonribosomal peptides	343.9004	0	232.216725	68.524925	ko00750	Fe_{SA}@AH	0.014214039	0.042642117	*
ko01055	Biosynthesis of vancomycin group antibiotics	44374.40375	30014.68375	25547.23	26474.6725	ko00750	blank	0.001045598	0.00627359	**
ko02010	ABC transporters	12653.36609	11021.93713	6953.825938	7007.265938	ko00760	Fe_{SA}@AH	0.002837545	0.008512635	**
ko02020	Two-component system	6558.698763	5997.558463	3441.412438	3511.635975	ko00760	blank	0.000586327	0.003517964	**
ko02030	Bacterial chemotaxis	24709.20951	21755.65576	7999.46415	8171.787988	ko00770	Fe_{SA}@AH	0.004729397	0.014188192	*
ko02040	Flagellar assembly	15030.60143	13592.29256	4241.680013	4900.9934	ko00770	blank	0.000785338	0.004712029	**
ko02060	Phosphotransferase system (PTS)	10965.03168	12629.06311	14051.6178	12200.87046	ko00785	Fe_{SA}@AH	0.016462014	0.049386043	*
ko03008	Ribosome biogenesis in eukaryotes	1057.1457	793.1971125	594.4023125	630.8616875	ko00785	blank	0.002380669	0.014284012	*
ko03010	Ribosome	32281.49259	23875.32159	17853.33511	18917.36244	ko00790	Fe_{SA}@AH	0.002837545	0.008512635	**
ko03013	RNA transport	1195.226263	861.2689875	605.3944125	668.4421875	ko00790	blank	0.000261158	0.001566947	**
ko03018	RNA degradation	11361.00839	8596.86345	6247.348025	6708.339	ko00860	blank	0.007698627	0.029208727	*
ko03020	RNA polymerase	21317.04633	15495.62629	9205.588425	10497.62313	ko00900	Fe_{SA}@AH	0.006051842	0.018155525	*
ko03030	DNA replication	24938.44549	18325.48683	14059.11236	14621.61778	ko00900	blank	0.005577994	0.018155525	*
ko03050	Proteasome	1.3750125	6.181	8.770825	7.2096375	ko00908	Fe_{SA}@AH	0.002380669	0.007142006	**
ko03060	Protein export	28821.70719	20771.77813	14794.03575	16131.196	ko00908	blank	0.000531195	0.003187171	**
ko03070	Bacterial secretion system	14264.48946	10077.89933	6762.339975	7453.511513	ko00910	Fe_{SA}@AH	0.003999392	0.011998177	*
ko03410	Base excision repair	18222.59196	13799.88298	10578.35934	11142.5941	ko00910	blank	0.000646749	0.003880495	**
ko03420	Nucleotide excision repair	16156.99996	11459.21729	8395.155788	8917.3753	ko00920	Fe_{SA}@AH	0.010515246	0.021030492	*
ko03430	Mismatch repair	34538.46375	25196.0269	18791.42523	19611.86659	ko00920	blank	0.001992081	0.011952486	*
ko03440	Homologous recombination	30396.67295	22632.40584	17540.73834	18275.84304	ko00960	Fe_{SA}@AH	0.001857108	0.011142645	*
ko03450	Non-homologous end-joining	128.39025	98.52375	47.87375	75.6665	ko00960	blank	0.009013924	0.027041772	*
ko04020	Calcium signaling pathway	10.7675375	2.1237	1.4583875	2.3278875	ko00970	Fe_{SA}@AH	0.007698627	0.023095882	*
ko04112	Cell cycle - Caulobacter	30299.34024	21757.07156	15115.64118	16368.61968	ko00970	blank	0.004350533	0.023095882	*

ko04122	Sulfur relay system	15897.93466	15748.94888	8781.906588	9655.186	ko01040	Fe_{SA}@AH	0.007698627	0.018018192	*
ko04141	Protein processing in endoplasmic reticulum	1078.827188	645.8223375	498.63915	570.757625	ko01040	blank	0.002178445	0.013070671	*
ko04146	Peroxisome	4212.012638	2585.669138	1232.769425	1511.061363	ko01055	Fe_{SA}@AH	0.006051842	0.018155525	*
ko04210	Apoptosis	1172.0764	449.6714875	283.6671125	414.1948	ko01055	blank	0.003373187	0.018155525	*
ko04512	ECM-receptor interaction	4.1607125	5.4330375	2.754475	3.2946375	ko02010	Fe_{SA}@AH	0.012241386	0.036724159	*
ko04621	NOD-like receptor signaling pathway	829.1041875	527.0400375	90.7576875	245.4236625	ko02010	blank	0.011349213	0.036724159	*
ko04626	Plant-pathogen interaction	3631.57605	3364.74145	2126.696888	2159.448025	ko02020	Fe_{SA}@AH	0.006051842	0.018155525	*
ko04910	Insulin signaling pathway	1454.555675	1362.151288	879.7493125	920.888325	ko02020	blank	0.006051842	0.018155525	*
ko04974	Protein digestion and absorption	1269.75255	361.02495	216.78555	326.33605	ko03008	Fe_{SA}@AH	0.007109759	0.021329277	*
						ko03008	blank	0.003094815	0.01856889	*
						ko03010	Fe_{SA}@AH	0.005577994	0.016733982	*
						ko03010	blank	0.003674177	0.016733982	*
						ko03013	Fe_{SA}@AH	0.014214039	0.042642117	*
						ko03013	blank	0.00656165	0.039369901	*
						ko03018	Fe_{SA}@AH	0.003373187	0.01011956	*
						ko03018	blank	0.001992081	0.01011956	*
						ko03020	Fe_{SA}@AH	0.005137883	0.015413649	*
						ko03020	blank	0.000435121	0.002610725	**
						ko03030	Fe_{SA}@AH	0.006051842	0.018155525	*
						ko03030	blank	0.003999392	0.018155525	*
						ko03060	Fe_{SA}@AH	0.004729397	0.014188192	*
						ko03060	blank	0.002599946	0.014188192	*
						ko03070	Fe_{SA}@AH	0.005577994	0.016733982	*
						ko03070	blank	0.002178445	0.013070671	*
						ko03410	Fe_{SA}@AH	0.013195152	0.039585457	*
						ko03410	blank	0.007109759	0.039585457	*
						ko03420	Fe_{SA}@AH	0.006051842	0.018155525	*
						ko03420	blank	0.003674177	0.018155525	*
						ko03430	Fe_{SA}@AH	0.007109759	0.021329277	*

	ko03430	blank	0.004350533	0.021329277	*
	ko03440	Fe_{SA}@AH	0.006051842	0.018155525	*
	ko03440	blank	0.003999392	0.018155525	*
	ko04020	blank	0.006950789	0.041704732	*
	ko04112	Fe_{SA}@AH	0.003373187	0.01011956	*
	ko04112	blank	0.001820454	0.01011956	*
	ko04122	blank	0.007698627	0.046191764	*
	ko04141	Fe_{SA}@AH	0.003674177	0.011022531	*
	ko04141	blank	0.000785338	0.004712029	**
	ko04141	AH	0.017698893	0.035397786	*
	ko04146	Fe_{SA}@AH	0.002599946	0.007799837	**
	ko04146	blank	0.000261158	0.001566947	**
	ko04210	blank	0.003999392	0.023996354	*
	ko04626	Fe_{SA}@AH	0.001992081	0.008512635	**
	ko04626	blank	0.002837545	0.008512635	**
	ko04974	blank	0.005137883	0.030827299	*

NOTES 1. Of any significant ($p<0.05$) changed (compared to PBS) microbial functions = 103.

NOTES 2. Of any very significant ($p<0.01$) changed (compared to PBS) microbial functions = 83.

Paired-wise Wilcoxon test with Bonferroni-Holm correction: * $p < 0.05$, ** $p < 0.01$ ($n=8$).

Gating strategy for flow cytometry

Gating of live cells

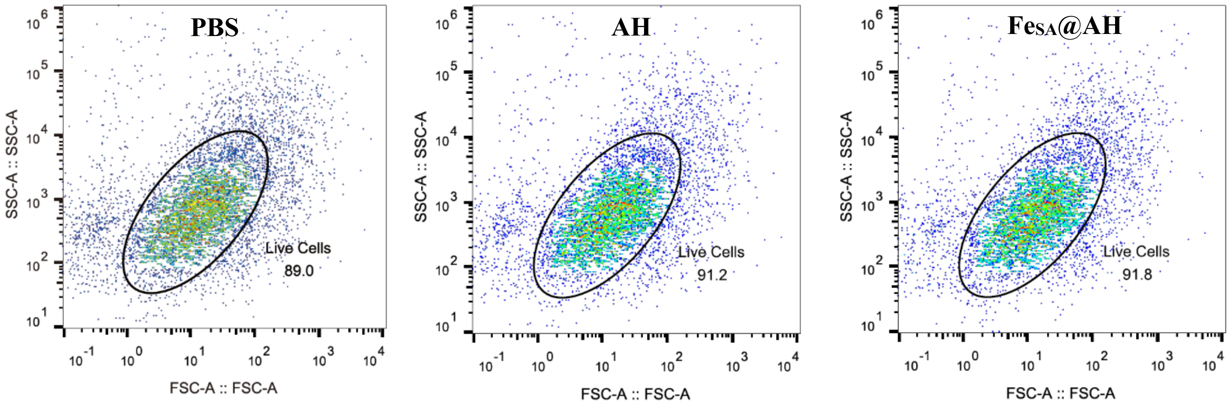

References

1. Liu, Y. et al. Biomimetic enzyme nanocomplexes and their use as antidotes and preventive measures for alcohol intoxication. *Nat. Nanotechnol.* **8**, 187-192 (2013).

2. Bagus, P.S. et al. Combined multiplet theory and experiment for the Fe 2p and 3p XPS of FeO and Fe(2)O(3). *J. Chem. Phys.* **154**, 094709 (2021).

3. Shen, Y. et al. Amyloid fibril systems reduce, stabilize and deliver bioavailable nanosized iron. *Nat. Nanotechnol.* **12**, 642-647 (2017).

4. Nelson, G.W., Perry, M., He, S.M., Zechel, D.L. & Horton, J.H. Characterization of covalently bonded proteins on poly(methyl methacrylate) by X-ray photoelectron spectroscopy. *Colloids Surf. B.* **78**, 61-68 (2010).

5. Vanea, E. & Simon, V. XPS study of protein adsorption onto nanocrystalline aluminosilicate microparticles. *Appl. Surf. Sci.* **257**, 2346-2352 (2011).

6. Lu, T., & Chen, Q. Independent gradient model based on Hirshfeld partition: A new method for visual study of interactions in chemical systems. *J. Comput. Chem.*, **43**, 539-555 (2022).
