# Supplementary material for: Single-site iron-anchored amyloid hydrogels as catalytic platforms for alcohol detoxification
Source: Nat Nanotechnol. 2024 May 13;19(8):1168–77. doi: 10.1038/s41565-024-01657-7 (PMC11329373; doi:10.1038/s41565-024-01657-7)
Supplement: Supplementary file 2 — Reporting Summary [file 41565_2024_1657_MOESM2_ESM.pdf]

nature portfolio

Corresponding author(s): Raffaele Mezzenga
Last updated by author(s): March 15th, 2024

Reporting Summary

Nature Portfolio wishes to improve the reproducibility of the work that we publish. This form provides structure for consistency and transparency in reporting. For further information on Nature Portfolio policies, see our Editorial Policies and the Editorial Policy Checklist.

Please do not complete any field with "not applicable" or n/a. Refer to the help text for what text to use if an item is not relevant to your study.
For final submission: please carefully check your responses for accuracy; you will not be able to make changes later.

Statistics

For all statistical analyses, confirm that the following items are present in the figure legend, table legend, main text, or Methods section.

n/a	Confirmed	
☐	☒	The exact sample size (*n*) for each experimental group/condition, given as a discrete number and unit of measurement
☐	☒	A statement on whether measurements were taken from distinct samples or whether the same sample was measured repeatedly
☐	☒	The statistical test(s) used AND whether they are one- or two-sided<br>*Only common tests should be described solely by name; describe more complex techniques in the Methods section.*
☐	☒	A description of all covariates tested
☒	☐	A description of any assumptions or corrections, such as tests of normality and adjustment for multiple comparisons
☒	☐	A full description of the statistical parameters including central tendency (e.g. means) or other basic estimates (e.g. regression coefficient) AND variation (e.g. standard deviation) or associated estimates of uncertainty (e.g. confidence intervals)
☐	☒	For null hypothesis testing, the test statistic (e.g. *F*, *t*, *r*) with confidence intervals, effect sizes, degrees of freedom and *P* value noted<br>*Give P values as exact values whenever suitable.*
☒	☐	For Bayesian analysis, information on the choice of priors and Markov chain Monte Carlo settings
☒	☐	For hierarchical and complex designs, identification of the appropriate level for tests and full reporting of outcomes
☒	☐	Estimates of effect sizes (e.g. Cohen's *d*, Pearson's *r*), indicating how they were calculated

Our web collection on statistics for biologists contains articles on many of the points above.

Software and code

Policy information about availability of computer code

Data collection	All of the all-atom MD simulations were performed on a GROMACS 2018 package.<br>The DFT calculations were performed using the CP2K software package (version 8.1).<br>For Intestinal flora analysis, in Taxa bar plots, and alpha- and beta-diversity analysis, were performed with the QIIME 2 (vision 2020.6) and R package (vision 3.6.3), and metabolic function was predicted using PICRUSt2.<br>Amplicon sequence variants (ASVs) were denoised and clustered by the UNOISE algorithm.
Data analysis	For the MD simulations, dynamic snapshot images were generated in Visual Molecular Dynamics (VMD) 1.9.3.<br>For the DFT calculations, the based isosurface maps were rendered by VMD from the cube files exported from Multiwfn 3.8.<br>For Intestinal flora analysis, the output file of metabolic function was analyzed using the STAMP software package (version 2.1.3).

For manuscripts utilizing custom algorithms or software that are central to the research but not yet described in published literature, software must be made available to editors and reviewers. We strongly encourage code deposition in a community repository (e.g. GitHub). See the Nature Portfolio guidelines for submitting code & software for further information.

Data

Policy information about availability of data

All manuscripts must include a data availability statement. This statement should provide the following information, where applicable:

- Accession codes, unique identifiers, or web links for publicly available datasets
- A description of any restrictions on data availability
- For clinical datasets or third party data, please ensure that the statement adheres to our policy

Source data are provided with this paper. Additional raw and analyzed datasets generated during this study are available for research purpose from the authors upon request. As R.M. and J.S. have declared a competing interest we prefer to be in a position to track those individuals using/accessing our data. Hence a data on request policy is more appropriate in this case.

Human research participants

Policy information about studies involving human research participants and Sex and Gender in Research.

Reporting on sex and gender	N/A
Population characteristics	N/A
Recruitment	N/A
Ethics oversight	N/A

Note that full information on the approval of the study protocol must also be provided in the manuscript.

Field-specific reporting

Please select the one below that is the best fit for your research. If you are not sure, read the appropriate sections before making your selection.

☒ Life sciences ☐ Behavioural & social sciences ☐ Ecological, evolutionary & environmental sciences

For a reference copy of the document with all sections, see nature.com/documents/nr-reporting-summary-flat.pdf

Life sciences study design

All studies must disclose on these points even when the disclosure is negative.

Sample size	No statistical methods were used to pre-determine sample sizes but our sample sizes are similar to that reported in a previous publication (Nature nanotechnology 8.3 (2013): 187-192).
Data exclusions	No sample was excluded from all analysis.
Replication	In our research, we performed experiments across at least 3 biological replicates, and findings were reproducible. Most of the experiments were replicated as stated in the figure legends. In terms of biological experiments, the reported data correspond to the main values derived from biological replicates including error. For images from microscopies, such as TEM, AFM, and so on, representative images were selected from at least six technical replicates.
Randomization	Samples and animals were randomized into different groups.
Blinding	Investigators were not blinded to group allocation.

Reporting for specific materials, systems and methods

We require information from authors about some types of materials, experimental systems and methods used in many studies. Here, indicate whether each material, system or method listed is relevant to your study. If you are not sure if a list item applies to your research, read the appropriate section before selecting a response.

Materials & experimental systems

n/a	Involved in the study
☒	☐ Antibodies
☒	☐ Eukaryotic cell lines
☒	☐ Palaeontology and archaeology
☐	☒ Animals and other organisms
☒	☐ Clinical data
☒	☐ Dual use research of concern

Methods

n/a	Involved in the study
☒	☐ ChIP-seq
☐	☒ Flow cytometry
☒	☐ MRI-based neuroimaging

Animals and other research organisms

Policy information about studies involving animals; ARRIVE guidelines recommended for reporting animal research, and Sex and Gender in Research

Laboratory animals	Male, wild type (WT) C57BL/6 mice aged 8-10 weeks, were purchased from Beijing Vital River Laboratory Animal Technology Co., Ltd. All animals were housed in SPF conditions at 23±1 °C with 50-60% humidity in a 12-hour light/dark cycle and were provided autoclaved water.
Wild animals	The study did not involved wild animals.
Reporting on sex	This information has not been collected.
Field-collected samples	This study did not involve samples collected from the field.
Ethics oversight	All of the murine experiments in the current study were approved by the Regulations of Beijing Laboratory Animal Management (approval number: AW40803202-5-1) and conducted according to the guidelines set forth in the Institutional Animal Care and Use Committee of China Agricultural University.

Note that full information on the approval of the study protocol must also be provided in the manuscript.

Flow Cytometry

Plots

Confirm that:

☒ The axis labels state the marker and fluorochrome used (e.g. CD4-FITC).

☒ The axis scales are clearly visible. Include numbers along axes only for bottom left plot of group (a 'group' is an analysis of identical markers).

☒ All plots are contour plots with outliers or pseudocolor plots.

☒ A numerical value for number of cells or percentage (with statistics) is provided.

Methodology

Sample preparation	Six-well plate seeded Caco-2 cells (2 mL and 3×105 cells/well) were treated with AH or Fe1@AH after 24 h of resuscitation under the following conditions: DMEM containing 10%(v/v) fetal bovine serum (FBS), nonessential amino acids, and 1% (v/v) penicillin–streptomycin (10,000 U/mL penicillin and 10 mg/mL streptomycin) at 37 °C in 5% CO2. The same amounts (4.0 g/mL, 40 μL) of AH or Fe1@AH were incubated with cells for another 24 h at 37 °C in 5% CO2. Cells treated with PBS were used as a negative control. After exposure, cells were washed three times in cold PBS, centrifuged at 1500rpm for 5min, and re-suspended in 500 μL of binding buffer. The cells were then Annexin V-FITC (Invitrogen™) stained.
Instrument	The cellular fluorescence was detected using a BD FACSCalibur 2 flow cytometer.
Software	BD FACSTM software for data collection.<br>FlowJo_v10.8.1 for data analysis.
Cell population abundance	At least 100,000 cells were measured for each sample.
Gating strategy	FSC vs. SSC gating to exclude debris. Boundaries for Gate 4 were based on the PBS-inoculated control.<br>Bottom right quadrant, FITC 9(+)/PI(−), early apoptotic cells; top right quadrant, FITC(+)/PI(+), necrotic cells; bottom lift quadrant, FITC(−)/PI(−), viable cells.

☒ Tick this box to confirm that a figure exemplifying the gating strategy is provided in the Supplementary Information.

This checklist template is licensed under a Creative Commons Attribution 4.0 International License, which permits use, sharing, adaptation, distribution and reproduction in any medium or format, as long as you give appropriate credit to the original author(s) and the source, provide a link to the Creative Commons license, and indicate if changes were made. The images or other third party material in this article are included in the article's Creative Commons license, unless indicated otherwise in a credit line to the material. If material is not included in the article's Creative Commons license and your intended use is not permitted by statutory regulation or exceeds the permitted use, you will need to obtain permission directly from the copyright holder. To view a copy of this license, visit http://creativecommons.org/licenses/by/4.0/
